# circRARS synergises with IGF2BP3 to regulate RNA methylation recognition to promote tumour progression in renal cell carcinoma

**DOI:** 10.1002/ctm2.1512

**Published:** 2023-12-11

**Authors:** Yuenan Liu, Kailei Chen, Yi Shou, Sen Li, Jun Wang, Qingyang Zhang, Ziwei Huang, Jiaju Xu, Mingfeng Li, Di Liu, Huageng Liang, Hongmei Yang, Xiaoping Zhang

**Affiliations:** ^1^ Department of Urology Union Hospital, Tongji Medical College Huazhong University of Science and Technology Wuhan P. R. China; ^2^ Institute of Urology Tongji Medical College Huazhong University of Science and Technology Wuhan P. R. China; ^3^ Department of Urology Sir Run Run Shaw Hospital Zhejiang University School of Medicine Hangzhou P. R. China; ^4^ Department of Biomedical Informatics Harvard Medical School Boston Massachusetts USA; ^5^ Department of Breast and Thyroid Surgery Union Hospital Tongji Medical College Huazhong University of Science and Technology Wuhan P. R. China; ^6^ Department of Pathogenic Biology School of Basic Medicine Huazhong University of Science and Technology Wuhan P. R. China; ^7^ Shenzhen Huazhong University of Science and Technology Research Institute Shenzhen P. R. China

**Keywords:** circRARS, IGF2BP3, m^6^A methylation, renal cell carcinoma, tumour progression

## Abstract

As the most prominent RNA modification, N6‐methyladenosine (m^6^A) participates in the regulation of tumour initiation and progression. Circular RNAs (circRNAs) also play crucial roles in ubiquitous life processes. Whether circRNAs are required for m^6^A regulation in renal cell carcinoma (RCC) remains unclear. Meta‐analysis and bioinformatics identified that IGF2BP3 was upregulated in RCC and indicated a worse prognosis. IGF2BP3 significantly promoted RCC progression in vitro and in vivo. Mechanistically, circRARS bound to KH1–KH2 domains of IGF2BP3 to enhance m^6^A modification recognition. A 12‐nt sequence (GUCUUCCAGCAA) was proven to be the IGF2BP3‐binding site of circRARS. Additionally, CAPN15, CD44, HMGA2, TNRC6A and ZMIZ2 were screened to be the target genes regulated by the IGF2BP3/circRARS complex in an m^6^A‐dependent manner. Stabiliser proteins, including HuR, Matrin3 and pAbPC1, were recruited by circRARS, thereby increasing the mRNA stability of the forementioned five target genes. Consequently, the IGF2BP3/circRARS complex facilitated the lipid accumulation of RCC cells and promoted sunitinib resistance via target genes. circRARS synergised with IGF2BP3 to facilitate m^6^A recognition, thereby promoting RCC progression. Thus, IGF2BP3 could be a potential biomarker for RCC diagnosis and prognosis and a therapeutic target.

## Graphical abstract

IGF2BP3 and circRARS interacted with each other through KH1–KH2 domains and a 12‐nt sequence (GUCUUCCAGCAA) to increase the m^6^A recognition of IGF2BP3. After m^6^A recognition, stabiliser proteins were recruited by circRARS, thereby increasing the mRNA stability of target genes, and promoting RCC progression. IGF2BP3 might be a novel therapeutic target for RCC.

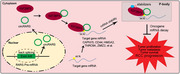

## 1 INTRODUCTION

Renal cell carcinoma (RCC) is one of the most common urinary malignancies with ascending global incidence.^1^ There will be 81 800 new diagnoses and 14 890 deaths from RCC in the USA in 2023.^2^ As the most prominent pathologic subtype of RCC, clear cell renal cell carcinoma (ccRCC) is characterised by high migratory ability, and resistance to radiotherapy and chemotherapy.^3^ And most advanced RCC patients develop resistance to targeted drugs such as sunitinib within 6–15 months.^4^, ^5^, ^6^ Thus, it is urgent to explore the mechanism of RCC progression, and infer effective biomarkers for early detection and prognosis prediction of RCC.

N6‐methyladenosine (m^6^A), the most prevalent internal modification in RNAs, is crucial in various malignancy‐related pathways.^7^, ^8^ However, the potential role of m^6^A in RCC progression remains an open question. m^6^A is regulated by writer, eraser and reader proteins.^9^, ^10^ Among them, the insulin‐like growth factor 2 mRNA‐binding proteins (IGF2BPs) family are important reader proteins. They contain several KH domains, which bind to RNA and regulate RNA metabolism. In particular, IGF2BP3 is significantly upregulated in RCC, recommended as a prognostic biomarker by multiple clinical studies.^11^ IGF2BP3 could recognise m^6^A‐modified sequence, regulating mRNA stability as well as translation.^12^ It has been demonstrated that with the assistance of long non‐coding RNAs (lncRNAs), IGF2BP3 stabilised CDK4 mRNA and drove RCC development through m^6^A modification.^13^ However, the underlying mechanism of IGF2BP3 recognition of m^6^A modification is still veiled. Whether there were other molecules involved in the regulation of m^6^A recognition of IGF2BP3 still needs to be explored.

Circular RNAs (circRNAs) are class of closed loop transcripts formed by precursor mRNAs.^14^ Compared to linear‐RNA, circRNAs are resistant to RNase digestion.^15^ With the development of RNA sequencing (RNA‐Seq), various circRNAs emerge as regulators in malignant cellular physiology.^14^, ^16^ The mechanism of how circRNAs regulate cellular function has been depicted with increasing sophistication and detail. The most prominent role circRNAs play is microRNA (miRNA) sponge. They bind miRNAs to attenuate their regulation on target genes.^17^ Other mechanisms include regulating transcription and splicing of mRNAs.^18^ CircRNAs also interact with RNA‐binding proteins^19^ and some of them can be translated into proteins.^20^

circRNAs interact with proteins via specific binding sites or tertiary structures.^21^ CircANRIL forms a stem‐loop structure to block interaction between PES1 and PeBoW complex.^22^ By binding proteins, circRNAs serve as protein sponges, protein decoys, protein scaffolds and protein recruiters. Quaking, an RNA‐binding protein, binds to sites flanking circRNA‐forming exons and regulates circRNAs formation.^23^ CircRNAs also function as decoys interacting with target proteins at specific cellular site, altering their biological function. CircAmotl1 binds and induces c‐Myc nuclear translocation, where it upregulates target genes, facilitating cell proliferation.^24^ As protein scaffolds, circRNAs enhance interaction between two or more binding proteins. For example, circFoxo3 binds to p53 and Mdm2, the E3 ubiquitin–protein ligase, inducing ubiquitination and degradation of p53.^25^ Moreover, it has been demonstrated that circRNAs could recruit proteins to certain cellular locations. CircAmotl1 recruits STAT3 from the cytoplasm to the nucleus and facilitates Dnmt3a transcription.^26^

Accumulating evidence shows that circRNAs could bind to m^6^A proteins and regulate their function directly. For example, circSTAG1 can bind to an m^6^A eraser, ALKBH5, and inhibit its nuclear translocation, thereby upregulating the m^6^A of FAAH mRNA.^27^ CircNDUFB2 was proven to enhance ubiquitination and degradation of IGF2BPs, suppressing lung cancer progression.^28^ Furthermore, it has been demonstrated that circPTPRA inhibits IGF2BP1 recognition of m^6^A sites in bladder cancer.^29^ Subsequently, are circRNAs involved in the m^6^A recognition of IGF2BP3 in RCC as well?

In this study, we validated the potential of IGF2BP3 as a biomarker for RCC diagnosis and prognosis through large‐scale meta‐analysis and bioinformatics. The mechanical excavation revealed that hsa_circ_0001550 (circRARS), a circRNA derived from exon2‐4 of arginyl‐tRNA synthetase 1 (RARS1), bound to KH1–KH2 domains of IGF2BP3 to enhance m^6^A modification recognition. A 12‐nt sequence (GUCUUCCAGCAA) was shown to be the IGF2BP3‐binding site of circRARS. And IGF2BP3/circRARS mediated the mRNA stability of CAPN15, CD44, HMGA2, TNRC6A and ZMIZ2 via an m^6^A‐dependent manner. Our results clarified the oncogenic function of IGF2BP3/circRARS complex in RCC progression and suggested a novel therapeutic target for RCC patients.

## 2 MATERIALS AND METHODS

### 2.1 Meta‐analysis

Articles about IGF2BP3 and RCC prognosis published before November 2020 were searched in database including PubMed, Web of Science, Embase and Cochrane Library. We used the following search strategy to filter studies on IGF2BP3 expression and RCC prognosis: (‘IGF2BP3’ or ‘insulin‐like growth factor 2 mRNA‐binding protein 3’ or ‘IMP3’), together with (‘outcome’ or ‘prognos*’ or ‘surviv*’) and (‘renal cell carcinoma’ or ‘RCC’ or ‘renal tumour’). Content filtering, data extraction and quality evaluation were performed independently by two authors (Y.L. and K.L.). Seven studies were finally included in this meta‐analysis (Table S1).^11^, ^30^, ^31^, ^32^, ^33^, ^34^, ^35^ Newcastle–Ottawa scale was used for quality assessment and score higher than 6 was set as inclusion threshold. Hazard ratio (HR) and relevant 95% confidence interval (CI) were calculated to assess the effect of high IGF2BP3 expression on the overall survival (OS) and disease‐free survival (DFS) of RCC patients. HR > 1 presented that higher IGF2BP3 expression indicated a shorter OS or DFS. Statistical heterogeneity was evaluated with Cochran's *Q*‐test and Higgins *I* ^2^ metrics. The cutoff value of obvious heterogeneity was set as *I* ^2^ > 50%.^36^ When no heterogeneity existed, fixed‐effect model could be applied and the random‐effect model was used in other cases.^37^ Due to the heterogeneities found, the random‐effect model was applied to all the meta‐analyses included in this study. As for bias analysis, we have done sensitivity analysis to evaluate the stability of pooled results and the funnel plots to estimate the publication bias.^38^ After all this assessment, the results of the meta‐analysis were solid and reliable. And all the calculation and analysis were performed with RevMan v5.3.

### 2.2 Human RCC tissue samples

Fifty pairs of RCC specimens and para‐cancer renal tissues from surgical resection were obtained from the authors’ institute. All the samples were frozen in −80°C immediately after surgery removal. Pathological diagnosis was confirmed. Informed consents were obtained from all patients preoperatively. This study was approved by the Ethic Committee of Human Research of HUST.

### 2.3 Cell culture

Human RCC cell lines (ACHN, A498, Caki‐1, OS‐RC‐2, 786‐O) and immortalised renal tubular epithelial cell HK‐2 were obtained from the American Type Culture Collection in the USA and cultured in Dulbecco's Modified Eagle's Medium high‐glucose (BI, Beit‐Haemek) supplemented with 10% foetal bovine serum (BI, Beit‐Haemek) and 1% streptomycin–penicillin (Google Biotechnology) at 37°C with 5% CO_2_.

### 2.4 Plasmid construction, transient transfection and lentivirus infection assay

The sequences of siRNA are listed in Table S2. IGF2BP3 cDNA was purchased from Genomeditech (Shanghai) and cloned into p3×FLAG‐CMV‐10 vector to construct the overexpression plasmid. circRARS wild‐type (circRARS‐WT) and circRARS‐M12 plasmid were synthesised and purchased from Genomeditech (Shanghai). Truncations of IGF2BP3 were amplified with primers, as shown in Table S3, and were cloned into p3×FLAG‐CMV‐10 vector. Lipofectamine 3000 reagent (Invitrogen) was applied following the manufacturer's protocols for transient transfection when RCC cells were at 50% confluence. Lentivirus (sg IGF2BP3 from GeneChem; circRARS from Genomeditech) was added into RCC cells at 40% confluence. An amount of 5 μg/mL puromycin or 5 μg/mL blasticidin was used to screen stable cells.

### 2.5 IGF2BP3 knockout in RCC cells

The sequences of sgRNA of IGF2BP3 are listed in Table S2. RCC cells were first seeded into the six‐well plate. Lentivirus with sgIGF2BP3 was added to RCC cells at 40% confluence. Forty‐eight hours later, 5 μg/mL puromycin was used for cell screening for 7 days. Stably infected RCC cells were then seeded into 96‐well plates for single clone selection. Western blot was performed for knockout validation.

### 2.6 Cell proliferation, migration and invasion assays

Cell counting kit 8 (CCK8, Beyotime) was applied to examine cell proliferation ability and sensitivity to sunitinib as previously described.^39^ EdU (Beyotime) was performed according to the manufacturer's instructions. The migration and invasion abilities were measured as previously described.^39^

### 2.7 Cell cycle analysis

Cell cycle analysis of RCC cells was performed using a FACS scan flow cytometer after incubation with reagents from a PI/RNase Staining Kit (Beyotime). Data were analysed using ModFit LT 2.0.

### 2.8 Oil red O stain and triglyceride examination

RCC cells fixed with 4% formalin were washed with phosphate‐buffered saline (PBS), and were stained with oil red for 30 min at room temperature. The excess oil red was removed with PBS before observation. A triglyceride assay kit (Jiancheng) was used to assess the triglyceride level of RCC cells as previously described.^40^

### 2.9 RNA isolation and quantitative reverse transcription polymerase chain reaction

Total RNA of RCC tissues and cells were isolated with Ultrapure RNA Kit (CWBIO) and reverse‐transcribed using a Superscript II reverse transcription kit (Vazyme). After that, SYBR‐Green reagent (Vazyme) was applied for quantitative reverse transcription polymerase chain reaction (qRT‐PCR; Applied Biosystems). Primers were purchased from TSINGKE (TSINGKE) as Table S4 showed.

### 2.10 RNA cellular fractionation assay

Nuclear and cytoplasmic RNA of RCC cells was isolated with a PARIS Kit (Invitrogen). GAPDH was employed as a cytoplasmic control, while U6 served as a nuclear control.

### 2.11 Fluorescence in situ hybridisation

Cy3‐labelled circRARS probe was purchased from Sangon (Sangon Biotech; Table S5). Cy3‐labelled 18S probe and Cy3‐labelled U6 probe were used for cytoplasm and nucleus control respectively (RiboBio). Nuclei were stained with DAPI. For RNA hybridisation, the fluorescence in situ hybridisation (FISH) kit from RiboBio (Guangzhou) was utilised.

### 2.12 RNA stability assay

At 60% confluency of RCC cells, 5 μg/mL actinomycin D or Dimethyl sulfoxide was added. Cells were collected after indicated time. After that, total RNA was isolated with miRNeasy Kit (Qiagen Inc.) and analysed by qRT‐PCR. U6 was used for normalisation.

### 2.13 Biotin‐labelled RNA pull‐down

Biotin‐labelled circRARS probe was purchased from Sangon (Sangon Biotech; Table S6). The biotinylated circRNA was pulled down by incubating the RCC cell lysates with streptavidin–agarose beads (Invitrogen). Precipitated proteins were separated using SDS‐PAGE.

### 2.14 Western blotting, immunohistochemistry and immunofluorescence

These experiments were performed as previously described.^40^ The antibodies information is shown in Table S7.

### 2.15 Co‐immunoprecipitation

RCC cells were lysed in IP lysis buffer (Beyotime) containing protease inhibitor (Takara). Following centrifugation at 12 000× *g* for 10 min, the supernatants of cell lysates were collected. Antibody against IGF2BP3 (1:100, Proteintech) was added into supernatants and upside down at 4°C overnight. Meanwhile, antibody against IgG (1:100, Abcam) was used as negative control. Then protein A/G magnetic beads (MCE) were added into supernatants and upside down at 4°C for 2 h. The immune‐complexes were separated using SDS‐PAGE.

### 2.16 Cross‐linking RNA‐binding protein immunoprecipitation

Totally 1 × 10^8^ RCC cells were cross‐linked with ultraviolet light (250 J/cm^2^) for 10 min in PBS and collected by scraping. The RNA‐binding protein immunoprecipitation (RIP) kit (Millipore) was utilised. Cross‐linked cells were lysed and antibodies against IGF2BP3, m^6^A or IgG were pre‐incubated with magnetic beads for 30 min. Then, magnetic beads and cell lysis were incubated together at 4°C overnight. RNA‐Seq or qRT‐PCR were performed for co‐precipitated RNAs detection (primers are shown in Table S8). The sequencing of co‐precipitated RNAs was performed and analysed by Novogene Bioinformatics Technology Co., Ltd.

### 2.17 RNA sequencing

Total RNA of RCC tissues or cells were extracted with TRIzol reagent (Beyotime). Illumina HiSeq X Ten platform was used to prepare library and to do the transcriptome sequencing. The sequencing and analysis were carried out by SeqHealth Technology Co., Ltd. The cutoff value was |fold change| > 2 and *p*‐value < .05.

### 2.18 Orthotopic renal tumour injection assays and tail‐vein tumour injection assays

The Animal Ethics Committee of HUST proved all in vivo experiments in this study. ACHN cells stably transfected with IGF2BP3 vector or si‐circRARS were used for injection. Four‐week‐old male BALB/c nude mice were purchased from HFK Biotechnology. For orthotopic renal tumour injection assays, mice were anesthetised. Left kidney was exposed from the left subcostal side. A total of 1 × 10^6^ ACHN cells was injected into left renal capsule (*n* = 5 mice/group). Then, we sutured the wound layer by layer. Tumour size was measured 4 weeks later with in‐Vivo FX PRO small animal imaging system (BRUKER). After that, we executed the mice and collected tumours for immunohistochemistry (IHC) assays and Western blotting. For metastasis model construction, 1 × 10^6^ ACHN cells were injected into tail‐vein of each mouse (*n* = 5 mice/group). Ten weeks later, we executed the mice and collected lungs and livers for hematoxylin and eosin (H&E) staining.

### 2.19 Bioinformatic analysis

The expression of genes including IGF2BP3, CAPN15, CD44 and other related genes, as well as clinical data were downloaded from The Cancer Genome Atlas database (TCGA, https://xenabrowser.net/heatmap/, KIRC dataset), GEO database (https://www.ncbi.nlm.nih.gov/geo/, GSE46699 and GSE53757) and Oncomine database (https://www.oncomine.org/, Gumz RENAL and Jones RENAL). The interaction between IGF2BP3 with circRARS was predicted by catRAPID (http://service.tartaglialab.com/page/catrapid_group) with the sequence of IGF2BP3 protein and circRARS nucleotide. Target genes related signalling pathways were analysed using the gene set enrichment analysis (GSEA; http://software.broadinstitute.org/gsea/index.jsp). Based on *p*‐value (<.05) and False Discovery Rate (FDR, <.25), we identified gene sets which were associated with target genes in TCGA‐KIRC dataset. Differentially expression genes were analysed with R ‘limma’ (version 3.42.2) package. Gene ontology analysis and plotting were performed using R ‘clusterProfiler’ (version 3.14.3) package. Potential m6A‐modified regions of target genes were predicted based on the IGF2BP3‐targeted RIP‐sequencing (RIP‐Seq) datasets and METTL3‐knockdown m^6^A‐targeted RIP‐Seq datasets (downloaded from GEO database, GSE196564) on IGV software.

### 2.20 Statistical analysis

Student's *t*‐test, analysis of variance, univariate and multivariate Cox regression analysis, *χ* ^2^ analysis, receiver operator characteristic (ROC) curve, etc., were utilised in this study as indicated. All data are shown as mean ± standard error of the mean (S.E.M.). Analyses were performed by GraphPad Prism 7.0 and SPSS 22.0. *p* < .05 was considered to be statistically significant (^*^ *p* < .05, ^**^ *p* < .01, ^***^ *p* < .001 and ^****^ *p* < .0001).

### 2.21 Availability of data and materials

The datasets supporting the conclusions of this manuscript are included within the manuscript and supporting information. Other data and materials about this study could be requested from the corresponding author.

## 3 RESULTS

### 3.1 IGF2BP3 is a potential biomarker for prognosis and diagnosis of RCC, and promotes RCC progression

**FIGURE 1:**
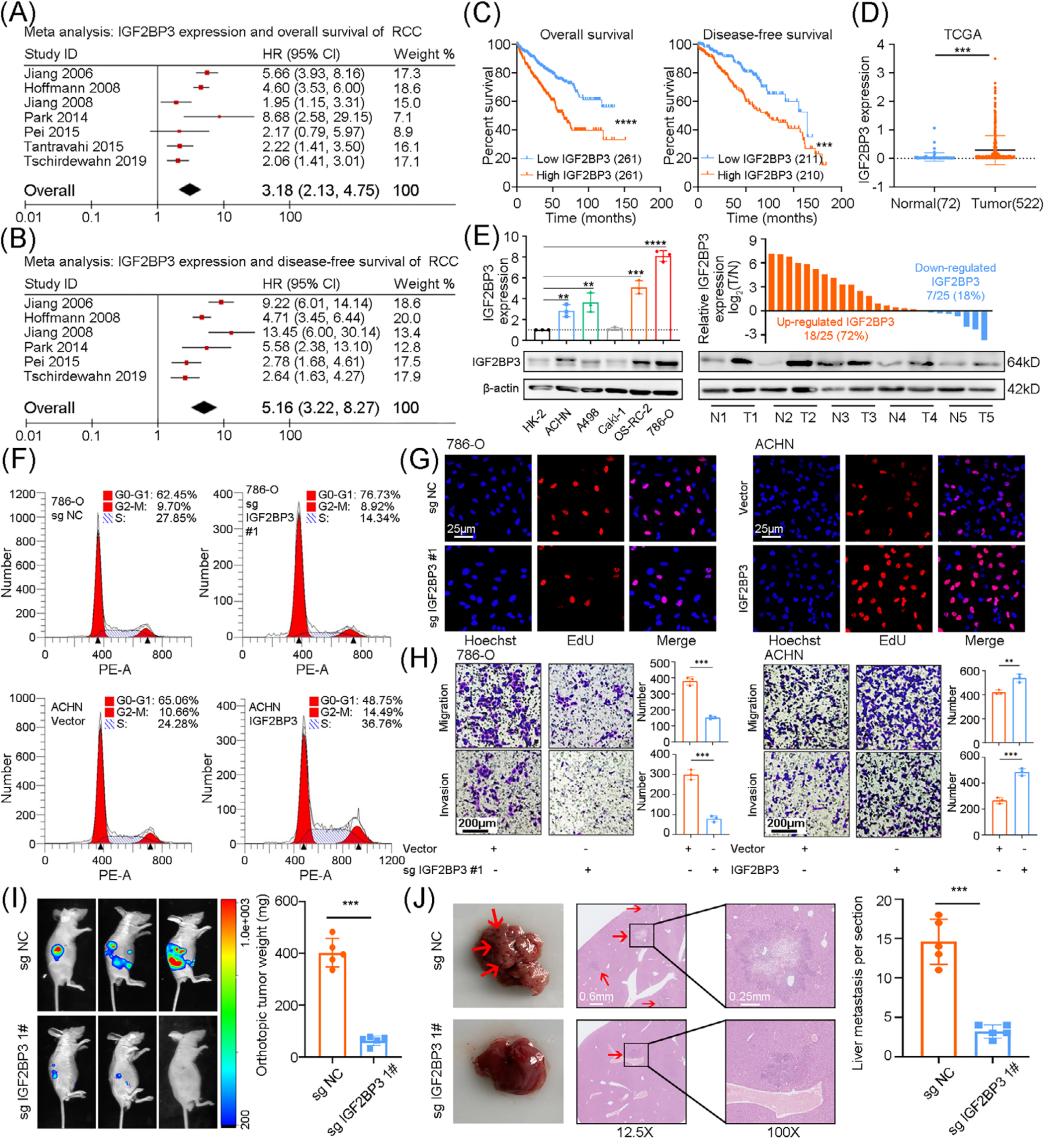
IGF2BP3 is a potential biomarker of RCC and promotes RCC progression. (A) Meta‐analysis indicated IGF2BP3 was a risk factor for RCC overall survival (OS, HR = 3.18, 95% CI = 2.13, 4.75). (B) Meta‐analysis revealed IGF2BP3 was a risk factor for RCC disease‐free survival (DFS, HR = 5.16, 95% CI = 3.22, 8.27). (C) The Kaplan–Meier survival analyses of IGF2BP3 in RCC for OS and DFS in TCGA. (D) The expression level of IGF2BP3 in 72 normal renal tissues and 522 RCC tissues from TCGA. (E) The mRNA and protein level of IGF2BP3 in RCC cell lines (ACHN, A498, Caki‐1, OS‐RC‐2 and 786‐O) and tissues. (F) Cell cycle analyses of RCC cells after IGF2BP3 knocked out or overexpressed using flow cytometry. (G) RCC cells proliferation was evaluated with EdU assays after IGF2BP3 knocked out or overexpressed. Scale bars, 25 μm. (H) Transwell assays were used to measure migration and invasion ability of RCC cells after transfection for 48 h. Scale bars, 200 μm. (I) Representative images and tumour weight of renal tumours by orthotopic injection of stably IGF2BP3 knocked out ACHN cells in nude mice (*n* = 5 for each group). (J) Representative images, H&E staining, and tumour number of liver metastasis tumours by tail‐vein injection of stably IGF2BP3 knocked out ACHN cells in nude mice (*n* = 5 for each group). Scale bars, .6 mm. ^*^ *p* < .05, ^**^ *p* < .01, ^***^ *p* < .001, ^****^ *p* < .0001. Data are represented as mean ± standard deviation (SD).

We conducted meta‐analyses including seven studies and confirmed that IGF2BP3 was a risk factor for prognosis of RCC patients (Figure 1A,B and Table S1). Next, we determined IGF2BP3 expression in TCGA‐KIRC cohort and demonstrated that IGF2BP3 mRNA expressions were significantly higher in RCC tissues and correlated with prognosis (Figure 1C,D and Tables S9 and S10). Furthermore, IGF2BP3 was also found overexpressed in RCC in both Oncomine and GEO dataset (Figure S1A,B). Its mRNA level was associated with clinical stage and pathological grade (Figure S1C and Table S11). Moreover, ROC curve predicted reliable diagnostic potential of IGF2BP3 (Figure S1D). Consistently, in RCC tissues and cells collected in this study, the elevation of IGF2BP3 was also validated (Figures 1E and S1E,F).

To further explore the biological properties of IGF2BP3 in RCC progression, we designed a CRISPR‐sgRNA or a plasmid vehicle to specifically regulate IGF2BP3 expression in RCC cell lines (Figure S2A). CCK8 and EdU assays identified IGF2BP3 as a proliferation accelerator in RCC cells (Figures 1G and S2B,D). Overexpression of IGF2BP3 strikingly facilitated the cell cycle shift from G0/G1 phase to S phase (Figures 1F and S2C). IGF2BP3 could promote migration and invasion in wound healing assays and transwell assays (Figures 1H and S2E,F). And we found IGF2BP3 increased RCC cancer stemness (Figure S3A). We also validated the oncogenesis role of IGF2BP3 in caki‐1 (Figure S3B–F).

In vivo, stable knockdown of IGF2BP3 attenuated renal orthotopic xenograft growth of RCC (Figures 1I and S3G). The metastatic model of nude mice showed that IGF2BP3 knockdown could suppress liver metastasis (Figure 1J). Since von Hippel–Lindau gene (VHL) mutation is considered to be the most prominent genomic feature of ccRCC. We detected pVHL and HIFα expression with overexpression or knockdown of IGF2BP3 (Figure S3H). Together, IGF2BP3 could be a biomarker for prognosis and diagnosis of RCC, and knockdown of IGF2BP3 inhibited the proliferation, invasion, and metastasis of RCC.

### 3.2 IGF2BP3 recognises the m^6^A modification sites and binds to circRARS

**FIGURE 2:**
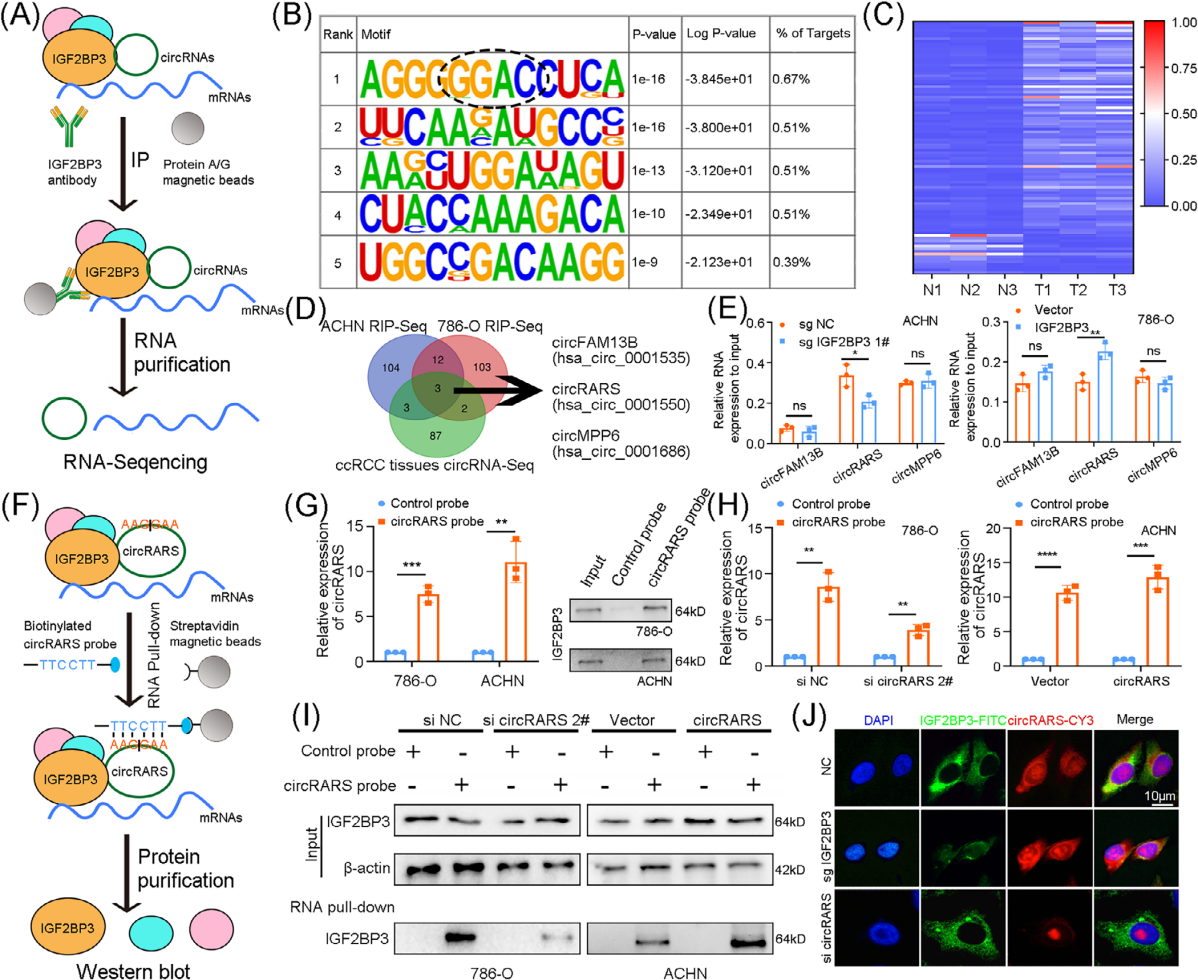
IGF2BP3 recognises the N6‐methyladenosine (m^6^A) modification sites and binds circRARS. (A) Schematic illustration of IGF2BP3‐targeted RIP sequencing system. (B) Top five binding sequences of IGF2BP3 in RCC cells analysed with HOMER motif analysis and GGAC, the m^6^A motif, was identified. (C) Heatmap of differentially expressed circRNAs in RCC tissues compared with adjacent normal tissues. (D) Overlap of IGF2BP3‐binding circRNAs screened by RIP sequencing and differentially expressed circRNAs from RCC tissues. (E) In IGF2BP3 knocked out or overexpressed RCC cells, RIP and qRT‐PCR were used to evaluate the combination between IGF2BP3 and circRNAs (circFAM13B, circRARS and circMPP6). (F) Schematic illustration of circRARS‐targeted RNA pulldown system. (G) qRT‐PCR was applied to assess the efficiency of the biotin‐labelled circRARS probe in the RNA pulldown system and Western blotting showed IGF2BP3 was pulled down by biotin‐labelled circRARS probe in lysates of RCC cells. (H) In RCC cells, the RNA pulldown efficiency of biotin‐labelled circRARS probe changed with circRARS knocked down or overexpressed. (I) Lysates of circRARS knocked down or overexpressed RCC cells were hybridised with biotin‐labelled circRARS probe and Western blotting showed the level of IGF2BP3 pulled down with circRARS probe. (J) Representative FISH and IF dual staining showed the expression level and localisation of IGF2BP3 (green) and circRARS (red) in 786‐O cells. Cell nuclear appeared in blue (DAPI). Scale bars, 10 μm. ^*^ *p* < .05, ^**^ *p* < .01, ^***^ *p* < .001, ^****^ *p* < .0001. Data are represented as mean ± standard deviation (SD).

IGF2BP3‐targeted RIP assays and RNA‐Seq were performed in 786‐O and ACHN cell lines (Figure 2A and Tables S12–S14). Efficiency of IGF2BP3 pull‐down was verified (Figure S4A). We identified the recognition ability of IGF2BP3 on m^6^A modification in RCC cell lines, as ‘GGAC’ motifs were mostly enriched in RIP samples, which is consistent with previous study (Figure 2B).^12^ To acquire the circRNAs involved in m^6^A recognition, the intersection of RIP‐Seq data of RCC cells and circRNA‐sequencing data of RCC tissues (with the cutoff of |fold change| ≥2 and *p*‐value ≤.05, Table S15) was obtained. CircFAM13B, circRARS and circMPP6 were filtered as potential binding circRNAs of IGF2BP3 in RCC cells (Figure 2C,D). Moreover, knockdown of IGF2BP3 attenuated the interaction between IGF2BP3 and circRARS, while overexpressed IGF2BP3 enhanced their interplay (Figures 2E and S4B). On the other hand, circRARS was knocked down or overexpressed with siRNA or plasmid (Figure S4C,D). circRARS probes were designed and verified (Figure 2F,G). Notably, circRARS knockdown decreased the level of IGF2BP3 that bound it, and vice versa (Figures 2H,I and S4E,F). Immunofluorescence (IF) and FISH assays showed co‐localisation of circRARS and IGF2BP3 in the cytoplasm of RCC cells (Figures 2J and S4G). Interestingly, circRARS and IGF2BP3 did not alter each other's expression (Figure S4H,I), implying that they might form an RNA–protein complex to function in RCC cells.

circRARS (hsa_circ_0001550) is a circRNA formed by reverse splicing of exons 2−4 of the RARS1 gene (Figure S5A). It could be amplified by the divergent primers in cDNA but not in genomic DNA (gDNA, Figure S5B). Compared with linear‐RARS, circRARS was more stable and more resistant to RNase R digestion (Figure S5C,D). Reverse transcription efficiency of circRARS was higher with random 6 than oligo dT primers, revealing that circRARS did not have poly(A) tail (Figure S5E). FISH assays indicated that circRARS was localised in the cytoplasm (Figure S5F,G). Confirmed by qRT‐PCR, circRARS was remarkably upregulated in RCC tissues and cell lines (Figure S6A). Knockdown or overexpression of circRARS significantly promoted or restrained RCC cells proliferation, cell cycle shift, migration and invasion in vitro (Figure S6B–E). Therefore, circRARS was considered to be an oncogene. Cell function rescue experiments were then performed. In overexpressed circRARS group, IGF2BP3 knockdown could largely suppress the progression of RCC cells compared to the sgNC/circRARS group. We also found that the oncogenic function of overexpressed IGF2BP3 could be rescued by silencing circRARS, indicating that IGF2BP3 synergises with circRARS to facilitate RCC progression (Figure S7A–C).

The epithelial–mesenchymal transition (EMT) is an essential pathway in many malignancies. We determined the EMT levels under different expressions of IGF2BP3 and circRARS. We found that IGF2BP3 and circRARS synergistically promote the EMT progress of RCC cells (Figure S8A).

In conclusion, we validated the potential of IGF2BP3 to bind m^6^A modification sites. And circRARS could bind to IGF2BP3, forming a complex to synergise the oncogenic properties in RCC cells.

### 3.3 circRARS interacted with KH1–KH2 domains of IGF2BP3 through its 12‐nt RNA sequence

**FIGURE 3:**
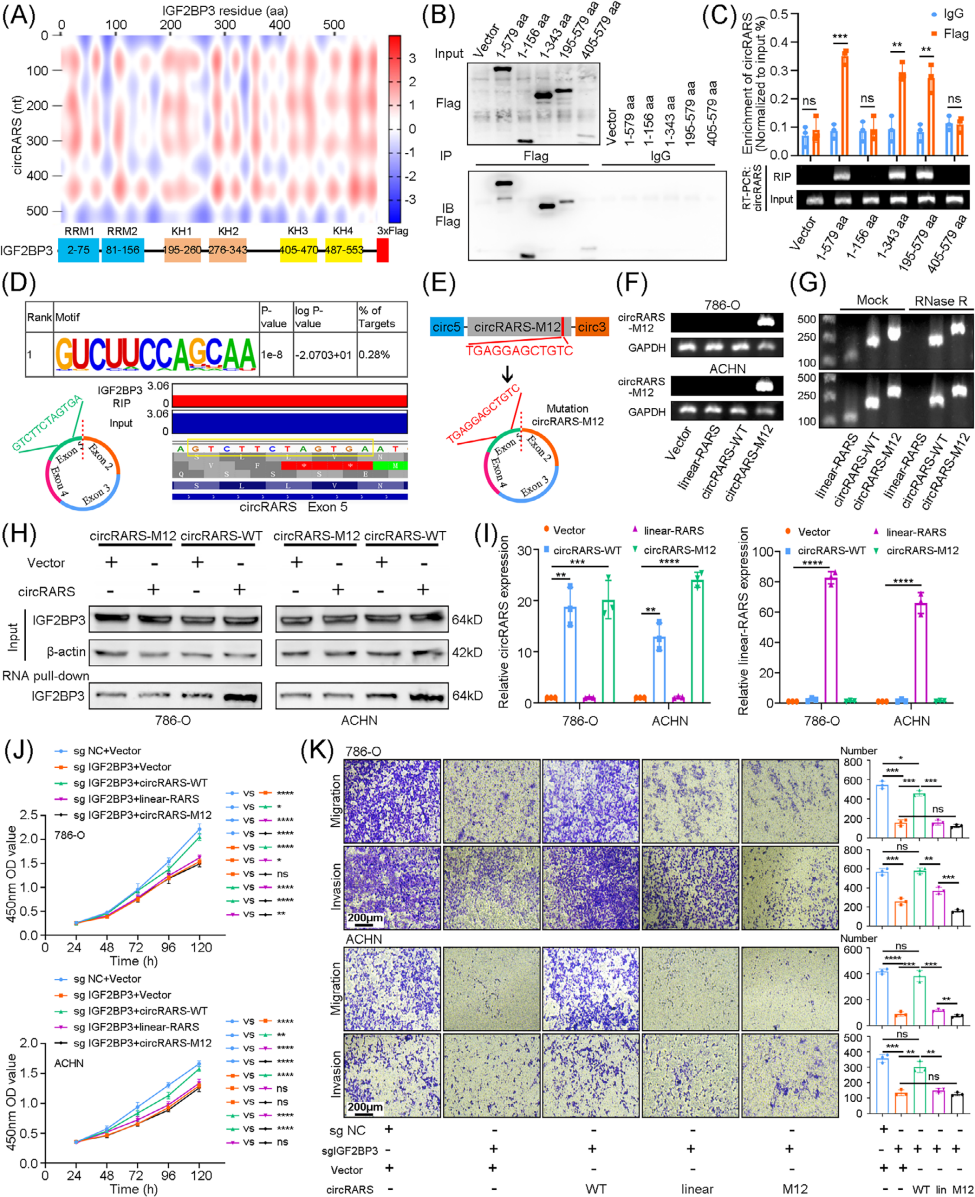
circRARS interacted with KH1–KH2 domains of IGF2BP3 through its 12‐nt RNA sequence. (A) catPAPID algorithm predicted the combination potential and sites between IGF2BP3 and circRARS. (B) Flag‐tagged IGF2BP3 full‐length or truncations vectors were constructed and transiently transfected into RCC cells and immunoprecipitation assays were conducted with anti‐Flag antibodies. (C) RIP assays showed circRARS was enriched in some of RCC cells that transiently transfected with Flag‐tagged IGF2BP3 full‐length or truncations vectors. (D) The most enriched binding sequence of IGF2BP3 in ACHN cells analysed by HOMER and this sequence were aligned to circRARS or RARS mRNA. (E) The structure of circRARS mutation plasmid circRARS‐M12 was showed. (F) qRT‐PCR with circRARS‐M12‐specific primers proved circRARS‐M12 expressed and circularised in RCC cells. (G) The relative RNA levels of linear‐RARS, circRARS and circRARS‐M12 were evaluated by qRT‐PCR in RCC cells treated with or without RNase R. (H) Lysates of RCC cells transfected with vector, wild‐type circRARS (circRARS‐WT) or circRARS‐M12 were hybridised with biotin‐labelled circRARS probe and Western blotting showed the level of IGF2BP3 pulled down with circRARS probe. (I) qRT‐PCR assays were applied to measure the expression level of circRARS or RARS mRNA in RCC cells after vector, linear‐RARS, circRARS‐WT or circRARS‐M12 transfection. (J) Proliferation ability of RCC cells co‐transfected with IGF2BP3 sgRNA and vector (or linear‐RARS, or circRARS‐WT, or circRARS‐M12) was determined using CCK8 assays. (K) Migration and invasion ability of RCC cells co‐transfected with IGF2BP3 sgRNA and vector (or linear‐RARS, or circRARS‐WT, or circRARS‐M12) was determined using transwell assays. Scale bars, 200 μm. ^*^ *p* < .05, ^**^ *p* < .01, ^***^ *p* < .001, ^****^ *p* < .0001. Data are represented as mean ± standard deviation (SD).

To explore the specific binding sites of IGF2BP3 and circRARS, catRAPID tools were applied to predict the probability and position of the interaction (Figure 3A). Next, flag‐tagged truncated plasmids of IGFBP3 were constructed. In vitro binding assay exhibited that KH1 and KH2 domains (195−343 amino acids) of FLAG‐tagged IGF2BP3 protein remarkably enhanced the interaction between IGF2BP3 and circRARS, which revealed that circRARS bound the KH1–KH2 domains of IGF2BP3 (Figure 3B,C). On the other hand, IGF2BP3 RIP‐Seq demonstrated that IGF2BP3 bound the ‘GUCUUCCAGCAA’ motif, which was exactly contained in circRARS sequence (Figure 3D). Subsequently, a circRARS‐WT and a circRARS plasmid containing the mutation of this 12‐nt sequence (circRARS‐M12) were constructed (Figure 3E). And we designed specific primers of circRARS‐M12 cross the junction site to make sure that only circRNA could be amplified. We transfected vector, linear‐RARS, circRARS‐WT and circRARS‐M12 plasmids into RCC cells, qRT‐PCR showed that only circRARS‐M12 plasmid could be detected (Figure 3F). Besides, circRARS‐WT and circRARS‐M12 both showed resistance against RNase R (Figure 3G). Thus, we confirmed circRARS‐M12 as a circular transcript molecule. Next, RNA pull‐down assays indicated that circRARS‐WT plasmid, but not circRARS‐M12 plasmid increased the level of IGF2BP3 that interaction with circRARS (Figure 3H). Furthermore, we explored the roles of circRARS‐M12 on RCC cells. Obviously, circRARS‐WT and circRARS‐M12 plasmids upregulated the expression of circRARS instead of RARS mRNA in RCC cells (Figure 3I). However, circRARS‐M12 failed to rescue the inhibition of proliferation, invasion and migration from IGF2BP3 knockdown (Figure 3J,K). In summary, circRARS bound to KH1–KH2 domains of IGF2BP3 through the ‘GTCTTCTAGTGA’ sequence. Mutation on this sequence could disrupt the IGF2BP3/circRARS complex formation and suppress its oncogenic function.

### 3.4 IGF2BP3/circRARS complex regulates downstream targets by altering RNA stabilities

**FIGURE 4:**
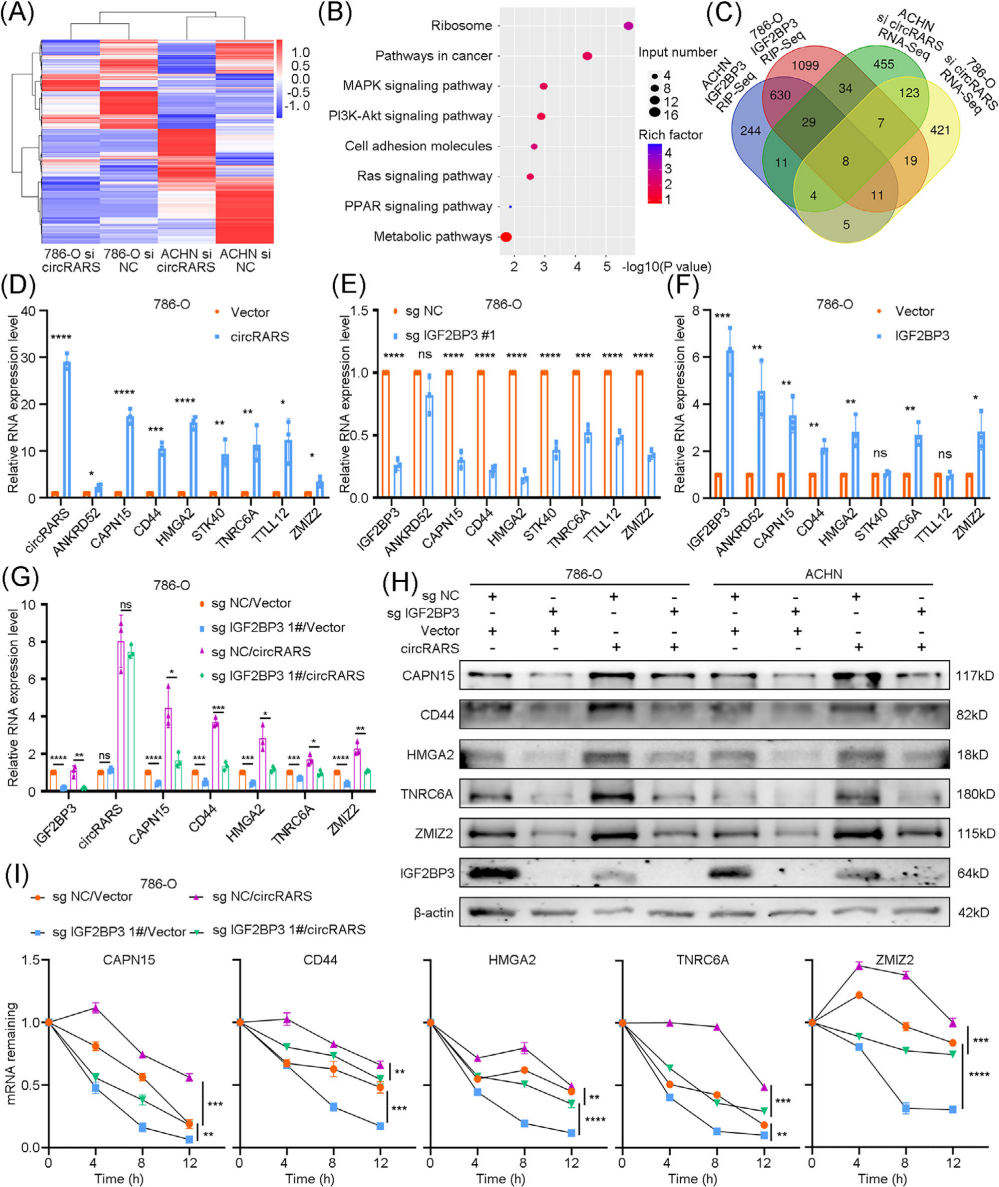
IGF2BP3/circRARS complex regulates downstream targets by altering RNA stabilities. (A) Heatmap showed the differentially expressed genes between circRARS knocked down and control 786‐O and ACHN cells. (B) Pathway enrichment analyses were analysed by using differentially expressed genes after circRARS knocked down. (C) Venn diagram uncovered the downstream genes regulated by IGF2BP3 and circRARS in RCC cells (filtered by |fold change| > 2 and *p*‐value < .05). (D) qRT‐PCR was used to examine the expression level of downstream genes in circRARS overexpression 786‐O cells. (E and F) qRT‐PCR was used to examine the expression level of downstream genes in IGF2BP3 knocked‐out (E) or overexpressed (F) 786‐O cells. (G) qRT‐PCR and (H) Western blotting showed the downstream genes expression level in RCC cells transfected with IGF2BP3 sgRNA or control, and co‐transfected with vector or circRARS plasmid. (I) The mRNA level of downstream genes were measured by qRT‐PCR in 786‐O cells after treatment with actinomycin D at the indicated time points. ^*^ *p* < .05, ^**^ *p* < .01, ^***^ *p* < .001, ^****^ *p* < .0001. Data are represented as mean ± standard deviation (SD).

To distinguish the downstream targets of IGF2BP3/circRARS complex, RNA‐Seq of circRARS knockdown RCC cells was performed (Figure 4A and Table S16). Several cancer‐related signalling pathways such as MAPK pathway and cell adhesion molecules were enriched (Figure 4B). With intersection of the circRARS knockdown RNA‐Seq and IGF2BP3‐targeted RIP‐Seq, eight potential downstream targets of IGF2BP3/circRARS complex were identified (Figure 4C). Their expression levels were verified by qRT‐PCR in circRARS overexpressed RCC cells (Figures 4D and S9A). Their mRNA levels were also evaluated in IGF2BP3 knockdown or overexpressed cells (Figures 4E,F and S9B,C). We found that the transcriptional levels of CAPN15, CD44, HMGA2, TNRC6A and ZMIZ2 were positively correlated with IGF2BP3/circRARS complex. Therefore, we speculated that the above five genes were regulated by IGF2BP3/circRARS complex. Rescue experiments confirmed the regulating effect of IGF2BP3/circRARS complex on them in both mRNA and protein levels (Figure 4G,H and S9D,E). IGF2BP3 could regulate downstream targets by altering mRNA stability. Thus, we utilised actinomycin D assay and found that IGF2BP3/circRARS complex could maintain the mRNA stability of CAPN15, CD44, HMGA2, TNRC6A and ZMIZ2 (Figures 4I and S9F).

### 3.5 IGF2BP3/circRARS complex regulates downstream targets in an m^6^A‐dependent manner

IGF2BP3 is known to regulate the stability of mRNA mainly via an m^6^A‐dependent manner. In this study, we utilised IGF2BP3‐targeted RIP‐Seq datasets and METTL3‐knockdown m^6^A‐targeted RIP‐Seq datasets on IGV software to identify potential m^6^A modification sites on the forementioned five genes.^41^ Notably, we found at least one IGF2BP3‐binding m^6^A modification site on each of the mRNA of CAPN15, CD44, HMGA2, TNRC6A and ZMIZ2 (Figure S10A–E).

**FIGURE 5:**
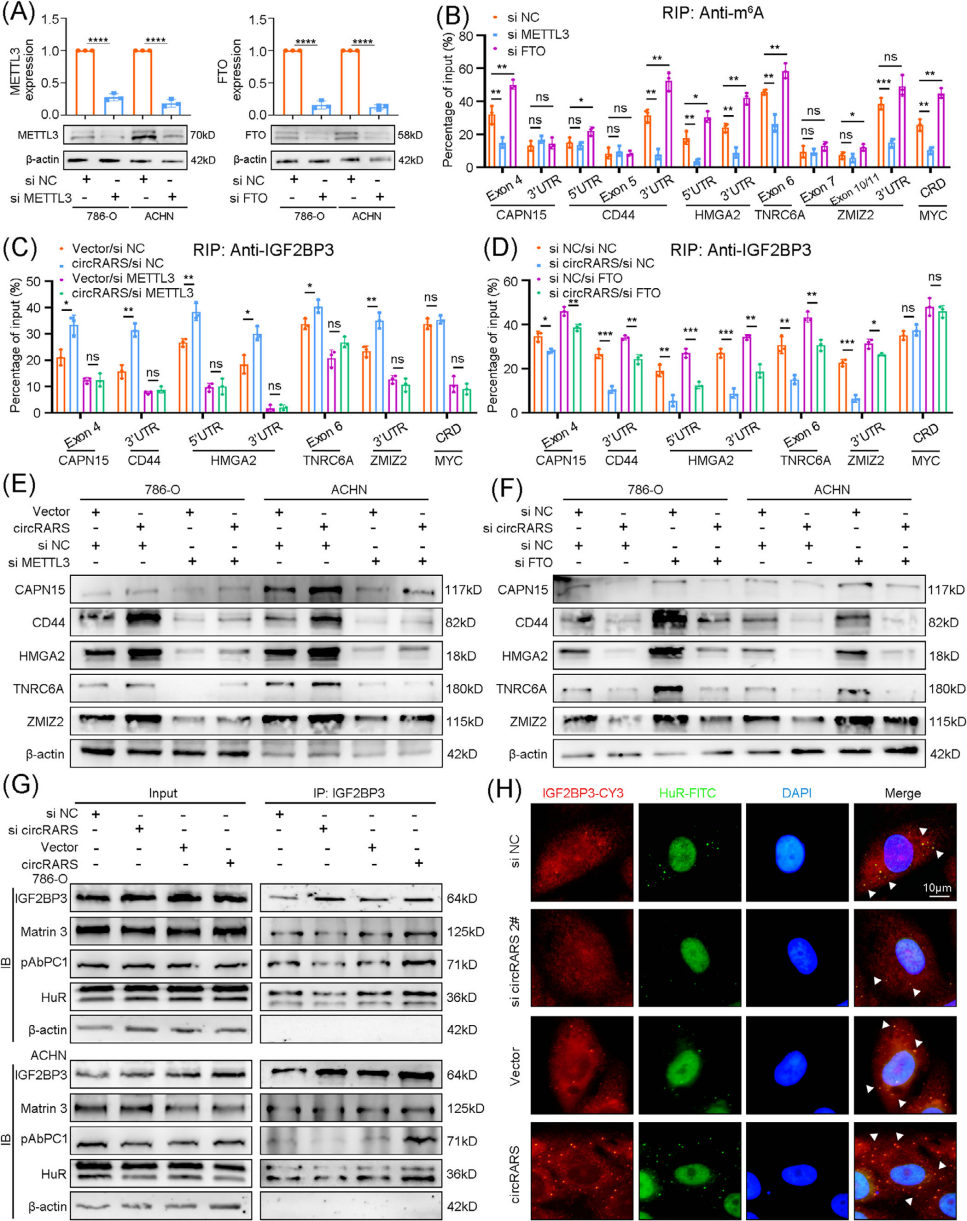
IGF2BP3/circRARS complex regulates downstream targets in an N6‐methyladenosine (m^6^A)‐dependent manner. (A) qRT‐PCR and Western blotting assays showed METTL3 and FTO expression levels in 786‐O and ACHN cells transfected with siRNA. (B) RIP‐targeted m^6^A and qRT‐PCR assays showed the enrichment regions of m^6^A modifications in the mRNA of CAPN15, CD44, HMGA2, TNRC6A and ZMIZ2 in METTL3 or FTO knocked down 786‐O cells. MYC CRD was used as a positive control. (C and D) RIP‐targeted IGF2BP3 and qRT‐PCR assays showed the combination ability between IGF2BP3 and m^6^A modification regions of downstream genes in circRARS overexpressed and METTL3 knocked down (C) or circRARS knocked down and FTO knocked down (D) 786‐O cells. (E and F) Western blotting assays showed the protein levels of CAPN15, CD44, HMGA2, TNRC6A and ZMIZ2 in circRARS overexpressed and METTL3 knocked down (E) or circRARS knocked down and FTO knocked down (F) 786‐O and ACHN cells. (G) Co‐IP assays demonstrated the binding of IGF2BP3 with stabilisers, including Matrin3, pAbPC1 and HuR, in circRARS knocked down or overexpressed 786‐O and ACHN cells. (H) Co‐localisation of IGF2BP proteins with HuR in circRARS knocked down or overexpressed 786‐O cells. Triangles indicated co‐localisation in cytoplasmic granules. Scale bars, 10 μm. ^*^ *p* < .05, ^**^ *p* < .01, ^***^ *p* < .001, ^****^ *p* < .0001. Data are represented as mean ± standard deviation (SD).

We then knocked down the expression of METTL3 or FTO to decrease or increase total m^6^A levels in RCC cells (Figure 5A). m^6^A‐RIP was performed to explore specific m^6^A‐binding sites, and exon 4 of CAPN15, 3′UTR of CD44, 5′UTR and 3′UTR of HMGA, exon 6 of TNRC6A and 3′UTR of ZMIZ2 were found (Figures 5B and S11A). Furthermore, IGF2BP3‐targeted RIP elucidated overexpression of circRARS facilitated interactions between IGF2BP3 and m^6^A modification sites on the mRNAs of downstream targets, which could be rescued by m^6^A inhibition (Figures 5C and S11B). Meanwhile, circRARS knockdown resulted in a reduction of IGF2BP3–m^6^A combination, which could be recovered by enhancing m^6^A modification with si‐FTO (Figures 5D and S11C). circRARS upregulated the protein levels of CAPN15, CD44, HMGA2, TNRC6A and ZMIZ2, whereas METTL3 or FTO knockdown reversed the alterations (Figure 5E,F).

Previous studies reported that after binding to m^6^A modification sites, IGF2BP3 interacted with stabiliser proteins and formed P‐bodies to stabilise mRNAs.^12^ Co‐immunoprecipitation (Co‐IP) assays revealed that knockdown or overexpression of circRARS altered the binding of IGF2BP3 to HuR, Martrin3 and pAbPC1 (Figure 5G). And circRARS knockdown reduced P‐bodies formed by IGF2BP3 and HuR, while circRARS overexpression increased the P‐bodies (Figures 5H and S11D). Therefore, circRARS facilitated IGF2BP3 to recognise m^6^A modification sites and to stabilise target mRNAs.

### 3.6 IGF2BP3/circRARS complex performs biological functions partially through downstream proteins

We analysed transcript levels of selected genes in TCGA–KIRC cohort and found the significantly high expression of CAPN15, CD44, TNRC6A and ZMIZ2 in RCC tissues. And CAPN15 and HMGA2 expression were positively correlated with IGF2BP3 (Figure S12A,D,G,J,M). Kaplan–Meier analyses revealed association between high expression of CD44, HMGA2, TNRC6A and ZMIZ2 and worse prognosis (Figure S12B,E,H,K,N). And expressions of all the five genes were associated with clinical characteristic of RCC patients (Figures S12C,F,I,L,O and S13A). Thus, we regarded them as oncogenes.

**FIGURE 6:**
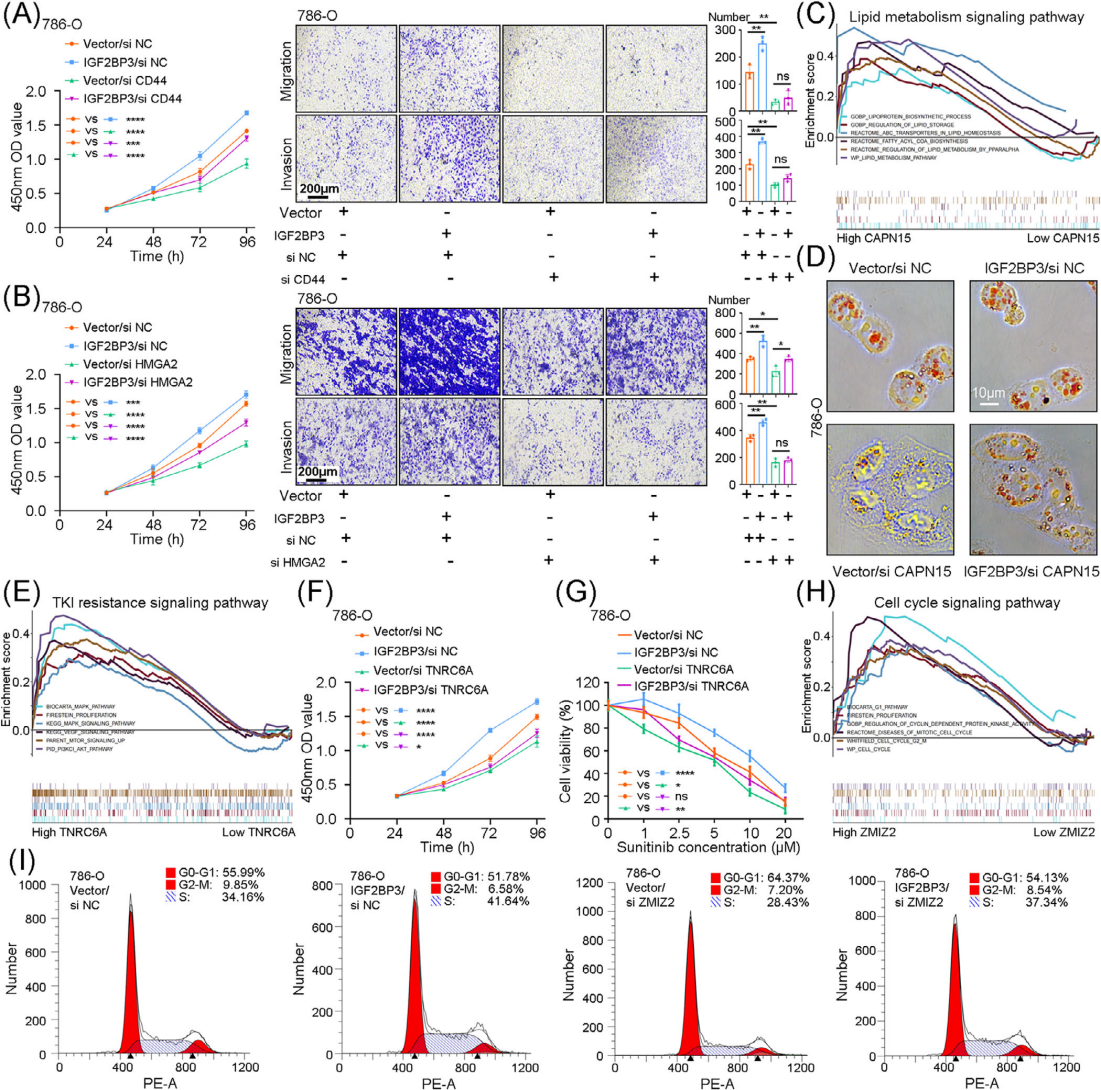
IGF2BP3/circRARS complex performs biological functions partially through downstream proteins. (A) Proliferation and metastasis were evaluated with CCK8 assays and transwell assays of IGF2BP3 overexpression 786‐O cells transiently transfected with CD44 siRNA or control. Scale bars, 200 μm. (B) Proliferation and metastasis were evaluated with CCK8 assays and transwell assays of IGF2BP3 overexpression 786‐O cells transiently transfected with HMGA2 siRNA or control. (C) GSEA for the correlations between lipid metabolism signalling pathway in RCC with the levels of the CAPN15 mRNA, according to TCGA–KIRC dataset. FDR <.25 and *p* <.05 were considered statistically significant. (D) Representative oil red O stains of IGF2BP3 overexpression 786‐O cells transiently transfected with CAPN15 siRNA or control. Scale bars, 10 μm. (E) GSEA for the correlations between TKI resistance signalling pathway in RCC with the levels of the TNRC6A mRNA, according to TCGA–KIRC dataset. FDR < .25 and *p* < .05 were considered statistically significant. (F) Proliferation was evaluated with CCK8 assays of IGF2BP3 overexpression 786‐O cells transiently transfected with TNRC6A siRNA or control. (G) Cell sensitivity to sunitinib was measured via CCK8 assays of IGF2BP3 overexpressed and TNRC6A knocked down 786‐O cells treated with sunitinib in different concentrations. (H) GSEA for the correlations between cell cycle signalling pathway in RCC with the levels of the ZMIZ2 mRNA, according to TCGA–KIRC dataset. FDR < .25 and *p* < .05 were considered statistically significant. (I) Cell cycle assays of IGF2BP3 overexpression 786‐O cells transiently transfected with ZMIZ2 siRNA or control. ^*^ *p* < .05, ^**^ *p* < .01, ^***^ *p* < .001, ^****^ *p* < .0001. Data are represented as mean ± standard deviation (SD).

It has been reported that IGF2BP3 could upregulate the expression of CD44 and HMGA2 and promote RCC proliferation and metastasis.^42^, ^43^ But the roles of CAPN15, TNRC6A and ZMIZ2 in tumourigenesis and their interaction with IGF2BP3 was not elucidated. We first interfered with the expression of them (Figure S14A,B). CCK8 and transwell assays suggested that IGF2BP3 promoted RCC proliferation and metastasis through CD44 and HMGA2 (Figures 6A,B and S15A,B), which was consistent with published studies. GSEA showed that CAPN15 was involved in various lipid metabolism pathways (Figure 6C). Oil red O assays and intracellular triglyceride test confirmed that CAPN15 knockdown partially rescued lipid accumulation induced by IGF2BP3 overexpression (Figures 6D and S15C,D). TNRC6A affected tyrosine kinase‐related signalling pathways, including MAPK, VEGF and mTOR (Figure 6E). Interestingly, TNRC6A knockdown partially reversed proliferation and sunitinib resistance of RCC cells induced by IGF2BP3 overexpression (Figures 6F,G and S15E,F). ZMIZ2 was significantly enriched in cell cycle signalling pathways (Figure 6H). Further analysis revealed that ZMIZ2 knockdown partially arrested the shift from G0/G1 phrase to S phrase induced by IGF2BP3 overexpression (Figures 6I and S15G). In summary, IGF2BP3/circRARS complex performed oncogenic functions partially through downstream molecules.

### 3.7 IGF2BP3/circRARS complex promotes RCC progression in vivo

**FIGURE 7:**
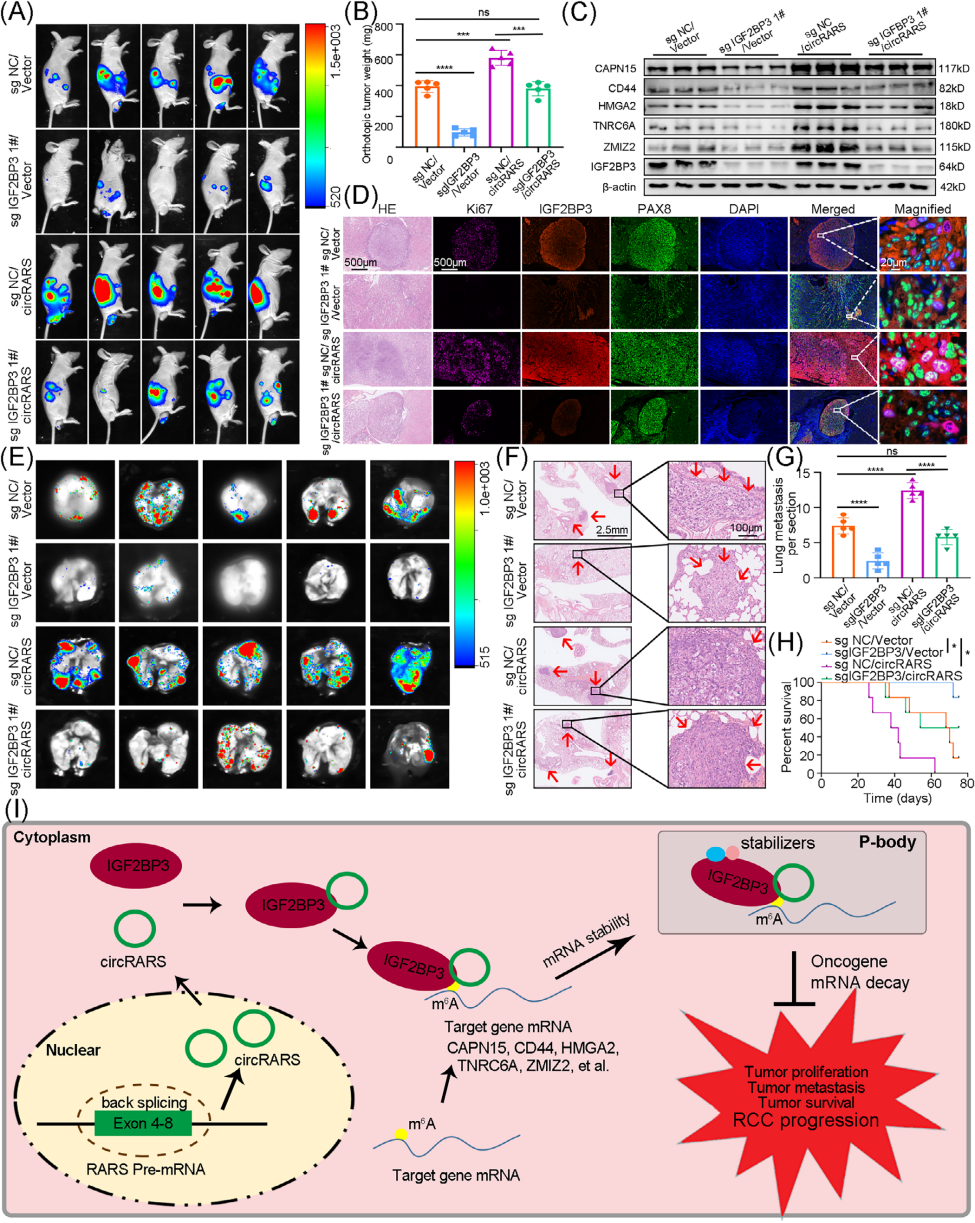
IGF2BP3/circRARS complex promotes RCC progression in vivo. (A) Representative in vivo bioluminescence images of the renal orthotopic xenografts growth of mice under various treatment conditions. (B) Weight of renal orthotopic xenografts from mice under various treatment conditions. (C) Protein expression of CAPN15, CD44, HMGA2, TNRC6A, ZMIZ2 and IGF2BP3 in renal orthotopic xenografts from mice under various treatment conditions showed by Western blotting. (D) Representative hematoxylin and eosin (H&E) stain and immunofluorescence (IF) images of renal orthotopic xenografts from mice under various treatment conditions. Violet, Ki67; red, IGF2BP3; green, PAX8; blue, DAPI. Scale bar, 500 μm. (E) Representative bioluminescence images of lung metastatic colonisation of nude mice with tail‐vein injection of ACHN cells under various treatment conditions (*n* = 5 for each group). (F) Representative H&E stain images of lung metastatic colonisation of nude mice with tail‐vein injection of ACHN cells. Scale bar, 2.5 mm. (G) Quantification of lung metastatic colonisation of nude mice with tail‐vein injection of ACHN cells. (H) Kaplan–Meier curves of nude mice with tail‐vein injection of ACHN cells. (I) Schematic diagram for the mechanisms of IGF2BP3/circRARS complex promoting RCC progression via an m^6^A‐dependent manner. ^*^ *p* < .05, ^**^ *p* < < .01, ^***^ *p* < .001, ^****^ *p* < .0001. Data are represented as mean ± standard deviation (SD).

ACHN cells stably transfected with IGF2BP3 sgRNA or control and circRARS or vector were injected into left renal capsule of 4‐week‐old nude mice for orthotopic xenograft models construction. Consistently, in vivo assays revealed that IGF2BP3 knockdown attenuated the RCC cancer stemness and inhibited orthotopic xenografts growth while overexpression of circRARS impaired the inhibition (Figures 7A,B and S16A). In orthotopic xenografts, Western blotting and IHC assays showed protein levels of CAPN15, CD44, HMGA2, TNRC6A and ZMIZ2 were regulated by IGF2BP3/circRARS complex (Figures 7C and S16B). IF showed that IGF2BP3/circRARS complex promoted proliferation of RCC cells. Ki67 was the biomarker of cell proliferation and PAX8 positive represented RCC cells (Figure 7D). Vein tail tumour injection assays were applied to show that IGF2BP3 knockdown diminished pulmonary metastasis while overexpression of circRARS facilitated pulmonary metastasis (Figure 7E). H&E stainings on lung of nude mice indicated that IGF2BP3/circRARS complex promoted the formation and proliferation of RCC pulmonary metastasis (Figure 7F,G). Finally, overexpression of IGF2BP3 could shorten survival time of nude mice, which could be rescued by circRARS knockdown (Figure 7H). Taken together, the oncogenic properties of IGF2BP3/circRARS complex in RCC were validated in vivo.

## 4 DISCUSSION

Emerging evidence demonstrated that m^6^A modification regulated RNA metabolism and played crucial roles in tumourigenesis, providing potential targets for diagnosis and precise treatment of cancers.^44^ IGF2BPs, a family of m^6^A‐related proteins, were proven with oncogenic properties in various malignancies. In breast cancer, IGF2BP1 stabilised the mRNA of c‐Myc to maintain the stemness of breast cancer cell.^45^ In melanoma, IGF2BP3 promoted metastasis in an m^6^A‐dependent manner.^46^ In RCC, IGF2BPs also acquired wide attention, represented by IGF2BP3. In 2006, a large‐scale clinical study reported IGF2BP3 as a biomarker for RCC metastasis and prognosis.^11^ Subsequently, multiple similar conclusions were drawn while the biological functions and molecular mechanisms were not elucidated until 2021. Gu et al. revealed that lncRNA DMDRMR bound IGF2BP3 to stabilise the mRNA of CDK4 and promoted proliferation of RCC cells.^13^ However, how IGF2BP3 binds to other m^6^A modification sites remains unclear. IGF2BPs mainly recognise the ‘GGAC’ sequence of RNA and more than 5000 mRNAs might be regulated by IGF2BPs in m^6^A‐dependent manners.^12^ In the current study, we verified that IGF2BP3 bound ‘GGAC’ sequence of downstream genes mRNA in RCC cells, which confirmed previous evidences.^12^, ^47^

Non‐coding RNAs included miRNAs, lncRNAs and circRNAs. Notably, due to their stability, non‐coding RNAs were likely to act as biomarkers for diagnosis and prognosis for cancer.^48^ It is widely accepted that there are vital regulatory mechanisms between m^6^A modification and non‐coding RNAs. On the one hand, the expression and biofunctions of non‐coding RNAs were influenced by m^6^A writer, eraser and reader proteins.^49^ For example, modified by m^6^A and recognised by YTHDF3, some circRNAs were reported to encode short peptides under the regulation of eukaryotic translation initiation factor 4 gamma 2 (eIF4G2), promoting tumour progression.^20^ On the other hand, non‐coding RNAs regulated m^6^A‐related proteins and influenced m^6^A modification as well. MiR‐33a, lncRNA GAS5 and circNDUFB2 were proved to regulate the expression of METTL3, ALKBH5 and IGF2BPs.^28^, ^50^, ^51^ And miRNAs bound to m^6^A sequences to regulate m^6^A modifications.^52^ Non‐coding RNAs could interact with reader proteins to recognise m^6^A modification sites.^13^, ^29^ To explore the roles of non‐coding RNAs in IGF2BP3‐m^6^A binding, we filtered multiple IGF2BP3‐binding circRNAs via RIP‐Seq. RNA pull‐down assays confirmed that circRARS bound to IGF2BP3 to form a complex. Thus, we hypothesised that circRARS regulated IGF2BP3 to recognise m^6^A sites. We then figured several downstream genes via RNA‐Seq of circRARS knockdown. In particular, the mRNA stabilities of CAPN15, CD44, HMGA2, TNRC6A and ZMIZ2 were regulated by IGF2BP3/circRARS complex via m^6^A‐dependent manners. However, the specific mechanisms in circRARS selective regulation required further explorations.

As an m^6^A reader protein, IGF2BPs mainly bound to m^6^A modification sites by their KH3–KH4 domains. And the KH1–KH2 domains acted as an accessory in the binding process. In this study, we confirmed that circRARS was mainly bound to KH1–KH2 domains of IGF2BP3 and presumed that circRARS could facilitate IGF2BP3 recognition of m^6^A sites. After combination with m^6^A modification sites, IGF2BP3 could recruit stabilisers including HuR, Matrin3 and pAbPC1, and formed a ‘safe house’ for mRNAs. HuR/IGF2BP3 interacted to form the P bodies and stabilised mRNA.^53^ IGF2BP3/HuR shuttled between ribosome and non‐ribosome fractions during stress condition and recovery, to regulate translation in stress response. In this study, we found circRARS was correlated with ribosome signalling pathway. Co‐IP and IF assays demonstrated that circRARS promoted IGF2BP3 recruitment of stabilisers proteins and co‐localisation of IGF2BP3 and HuR. Briefly, circRARS accelerated IGF2BP3 to act as a ‘safe house’, stabilising mRNAs and activating translation.

As previously mentioned, we found that the expression of CAPN15, CD44, HMGA2, TNRC6A and ZMIZ2 were regulated by IGF2BP3/circRARS complex. The canonical downstream targets of IGF2BP3 included Myc, CD44, HMGA2, etc. It has been reported that IGF2BP3 could bind to the CRD sequence of Myc.^12^ However, no correlation between Myc and IGF2BP3/circRARS was observed in this present study, probably because of a lack of pairing sequence on circRARS and Myc. In Hela cell, IGF2BP3 was reported to bind to 3′UTR and stabilise the mRNA of CD44.^43^ In hepatocarcinoma, lung cancer, and melanoma, IGF2BP3 maintained the stability of HMGA2 mRNA.^54^, ^55^, ^56^ When it comes to CAPN15, TNRC6A and ZMIZ2, there are few relevant studies yet. Calpains (CAPNs) are a family of genes participating in multiple cancer‐related bioprocesses including EMT.^57^ However, CAPN15's role in cancers remains unclear. We confirmed that CAPN15 facilitated lipid accumulation via intracellular triglyceride analysis and oil red O assays. TNRC6A, an RNA‐binding protein, could bind to miRNAs and form RNAi complexes, inhibiting the transcription of certain target mRNAs.^58^, ^59^ In RCC, TNRC6A altered the mRNA stability of HIF1α via P bodies formation.^53^ We explored that TNRC6A regulated the VEGFR‐related signalling pathways and mediated the TKIs‐resistance of RCC. The oncogenic roles of ZMIZ2 have been proposed before.^60^, ^61^ For example, ZMIZ2 promoted tumour proliferation of colon cancer by recruiting USP7, deubiquitinating β‐catenin, and thereby activating WNT signalling pathway.^60^ However, the underlying oncogenic mechanisms of CAPN15, TNRC6A and ZMIZ2 remain to be further elucidated.

Currently, m^6^A‐related proteins and non‐coding RNAs are regarded as potential diagnostic and prognostic ‘sentinels’ for cancers and therapeutic targets.^48^ This study demonstrated that IGF2BP3/circRARS complex promoted RCC progression in an m^6^A‐dependent manner. Targeting IGF2BP3/circRARS complex might provide us new strategies and directions for RCC patients management.

## AUTHOR CONTRIBUTIONS

Yuenan Liu, Kailei Chen and Yi Shou performed most of the experiments and wrote this manuscript and contributed equally to this work. Qingyang Zhang contributed extraordinary in the revision process and language polishing. Sen Li, Jun Wang and Ziwei Huang finished some of animal experiments and analysed the data. Jiaju Xu and Mingfeng Li conducted some of cell experiments and analysed the data. Di Liu and Huageng Liang collected RCC tissues and accomplished bioinformatic analysis. Hongmei Yang and Xiaoping Zhang designed the whole project and supervised all experiments. All authors revised and approved the manuscript.

## CONFLICT OF INTEREST STATEMENT

The authors declare they have no conflicts of interest.

## ETHICS STATEMENT

This study was approved by the Ethic Committee of Human Research of HUST and the Animal Ethics Committee of HUST.

## Supporting information

Supporting Information

Supporting Information

Supporting Information

Supporting Information

Supporting Information

Supporting Information

Supporting Information

Supporting Information

Supporting Information

Supporting Information

Supporting Information

Supporting Information

Supporting Information

Supporting Information

Supporting Information

Supporting Information

Supporting Information

## Data Availability

The datasets supporting the conclusions of this manuscript are included within the manuscript and supporting information. Other data and materials about this study could be requested from the corresponding author.
