# Supplementary material for: circRARS synergises with IGF2BP3 to regulate RNA methylation recognition to promote tumour progression in renal cell carcinoma
Source: Clin Transl Med. 2023 Dec 11;13(12):e1512. doi: 10.1002/ctm2.1512 (PMC10711645; doi:10.1002/ctm2.1512)
Supplement: Supplementary file 1 — Supporting Information [file CTM2-13-e1512-s010.docx]

**Figure S1.** IGF2BP3 is up-regulated in RCC and correlated with clinicopathological characteristics.


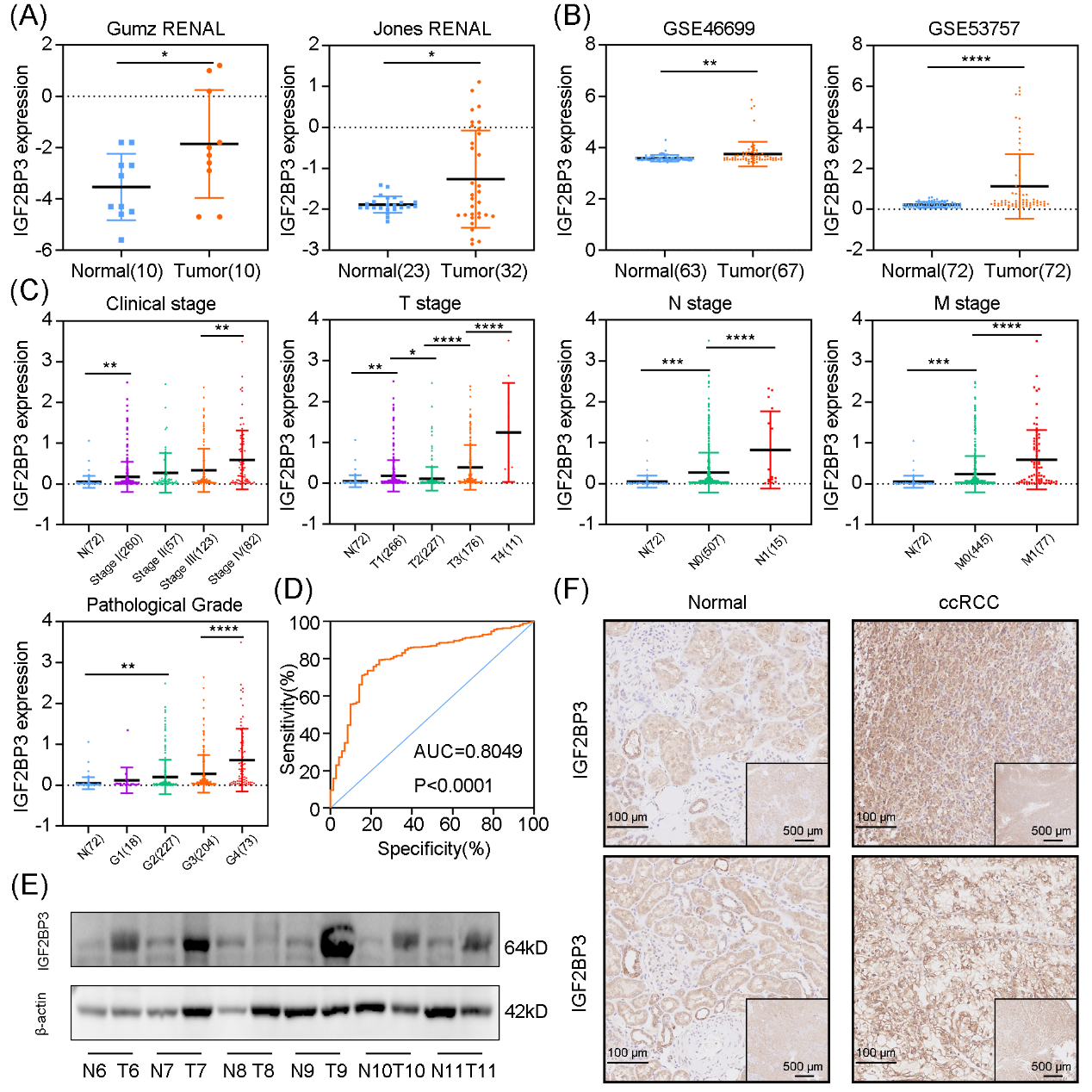


**Figure S1** IGF2BP3 is up-regulated in RCC and correlated with clinicopathological characteristics. **（A）** The expression level of IGF2BP3 in normal renal tissues and ccRCC tissues from Oncomine database (Gumz Renal and Jones Renal). **(B)** The expression level of IGF2BP3 in normal renal tissues and ccRCC tissues from GEO database (GSE46699 and GSE53757). **(C)** In TCGA-KIRC dataset, the expression of IGF2BP3 was related with various clinicopathological factors, including clinical stage, T stage, N stage, M stage, and pathological grade. **(D)** The receiver operating characteristic (ROC) curves of IGF2BP3 (AUC = 0.8049; p < 0.0001). **(E)** Western blotting assays showed the expression level of IGF2BP3 in ccRCC tissues and adjacent normal tissues. **(F)** IHC for IGF2BP3 in ccRCC tissues and paired normal tissues. Scale bars, 100 μm. *: P < 0.05, **: P < 0.01, ***P: < 0.001, ****P: < 0.0001. Error bars indicate mean ±SD.

**Figure S2.** IGF2BP3 promotes RCC progression *in vitro* and *in vivo*.


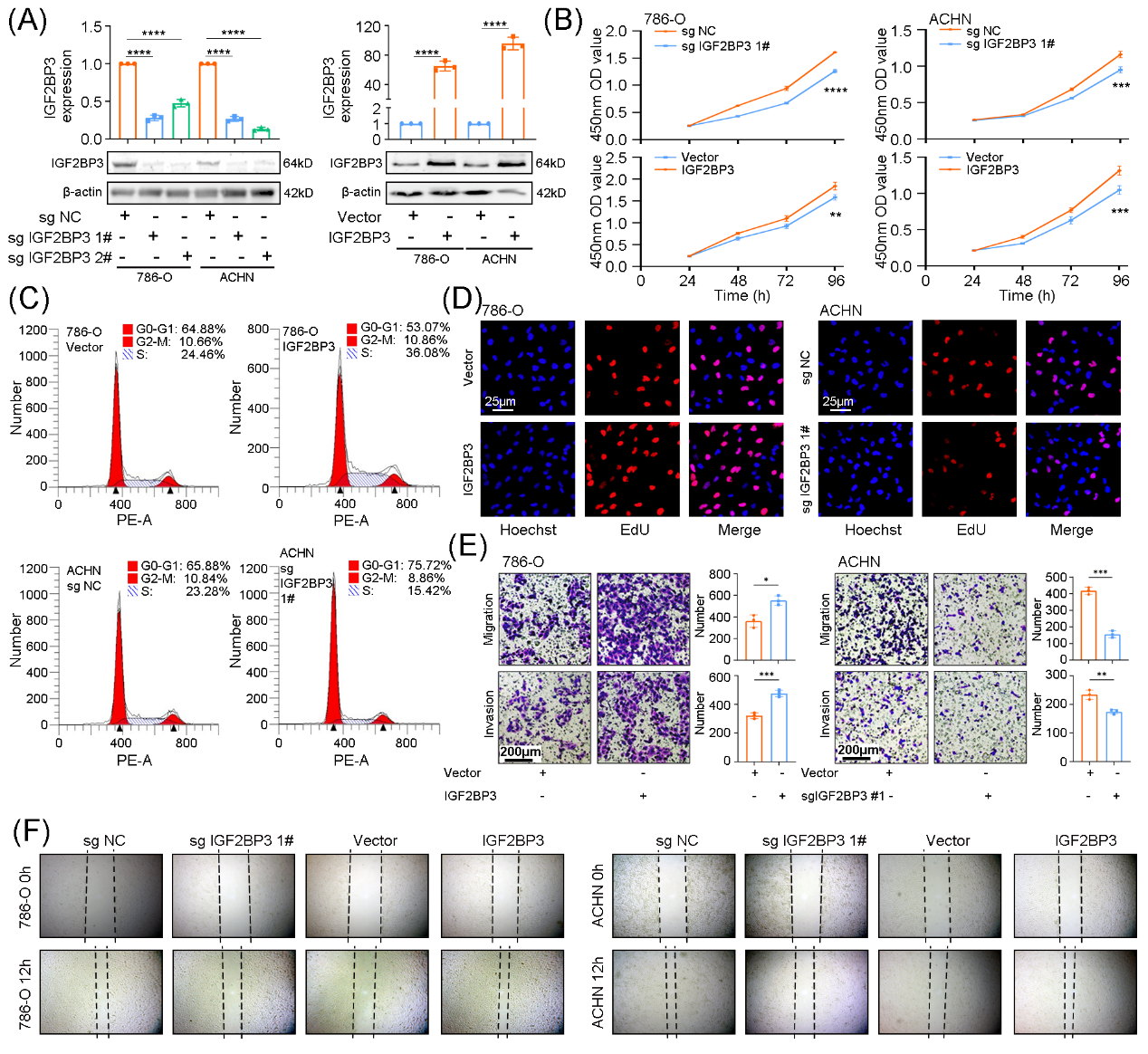


**Figure S2** IGF2BP3 promotes RCC progression *in vitro* and *in vivo*. **(A)** qRT-PCR and Western blotting assays were applied to analyze the expression level of IGF2BP3 after transfection with sg-RNA or overexpression vector for 48 h in RCC cells. **(B)** RCC cells proliferation was evaluated with CCK8 assays after IGF2BP3 knocked out or overexpressed. **(C)** Cell cycle assays of RCC cells with IGF2BP3 knocked out or overexpressed. **(D)** RCC cells proliferation was evaluated with EdU assays after IGF2BP3 knocked out or overexpressed. Scale bars, 25 μm. **(E-F)** Cell migration and invasion ability of IGF2BP3 knocked out or overexpressed RCC cells was measured with Transwell assays **(E)** or wound healing assays **(F)**. Scale bars, 200 μm. Magnification: x40 for wound healing assays. *: P < 0.05, **: P < 0.01, ***P: < 0.001, ****P: < 0.0001. Error bars indicate mean ±SD.

**Figure S3** IGF2BP3 promotes RCC progression *in vitro* and *in vivo* independently of pVHL.


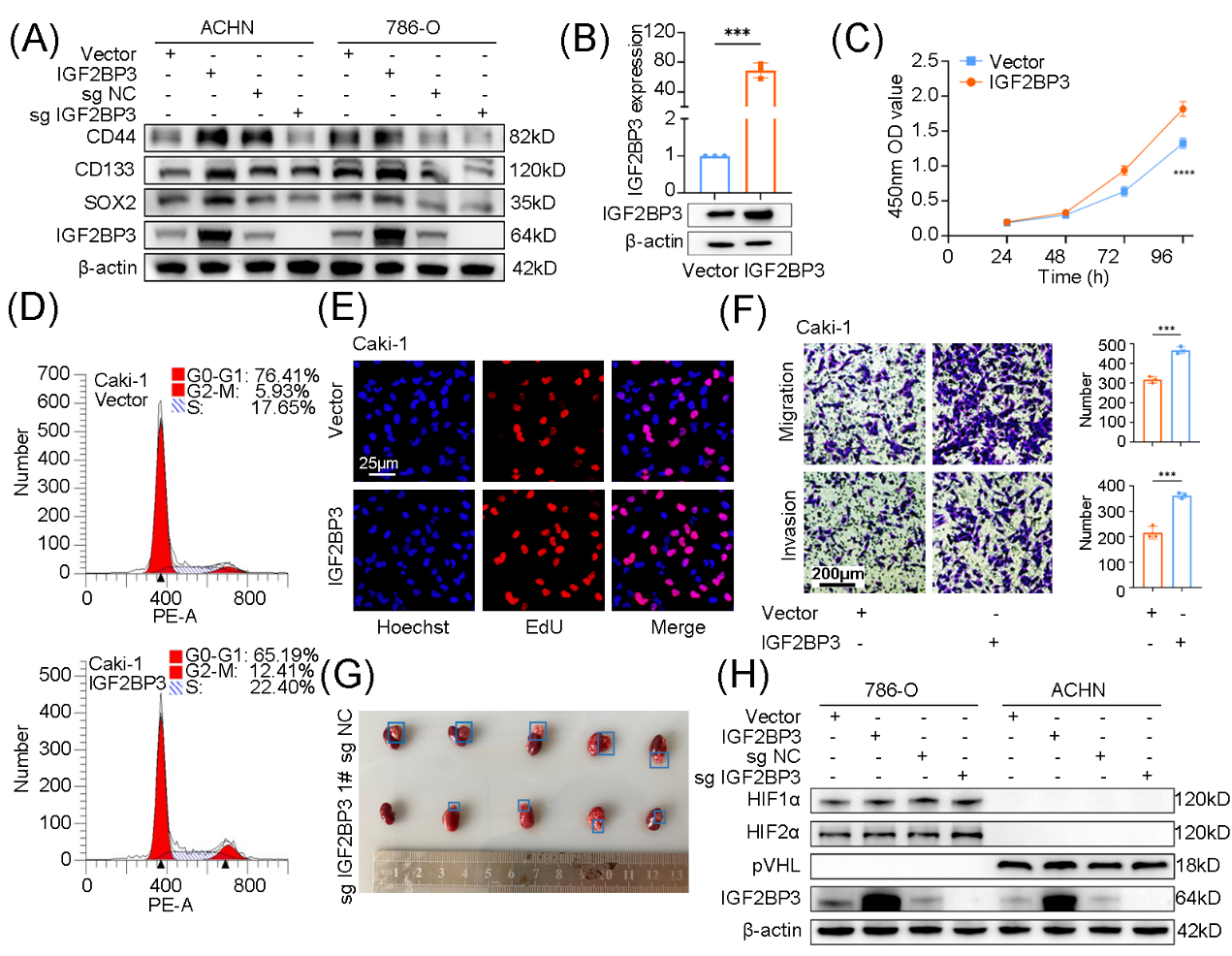


**Figure S3** IGF2BP3 promotes RCC progression *in vitro* and *in vivo* independently of pVHL. **(A)** Western blotting assays showed the expression of CD44, CD133, and SOX2 in IGF2BP3 knocked down or overexpressed RCC cells. **(B)** qRT-PCR and Western blotting assays were applied to analyze the expression level of IGF2BP3 after transfection with overexpression vector for 48 h in Caki-1 cells. **(C)** Caki-1 cells proliferation was evaluated with CCK8 assays after IGF2BP3 overexpressed. **(D)** Cell cycle assays of Caki-1 cells with IGF2BP3 overexpressed. **(E)** Caki-1 cells proliferation was evaluated with EdU assays after IGF2BP3 overexpressed. Scale bars, 25 μm. **(F)** Cell migration and invasion ability of IGF2BP3 overexpressed Caki-1 cells was measured with Transwell assays. Scale bars, 200 μm. **(G)** Images of renal orthotopic xenografts in nude mice with IGF2BP3 knocked out or control ACHN cells renal capsule injection. **(H)** Western blotting assays showed the expression of HIF1α, HIF2α and pVHL in IGF2BP3 knocked down or overexpressed RCC cells. *: P < 0.05, **: P < 0.01, ***P: < 0.001, ****P: < 0.0001. Error bars indicate mean ±SD.

**Figure S4.** IGF2BP3 binds circRARS to form a complex.


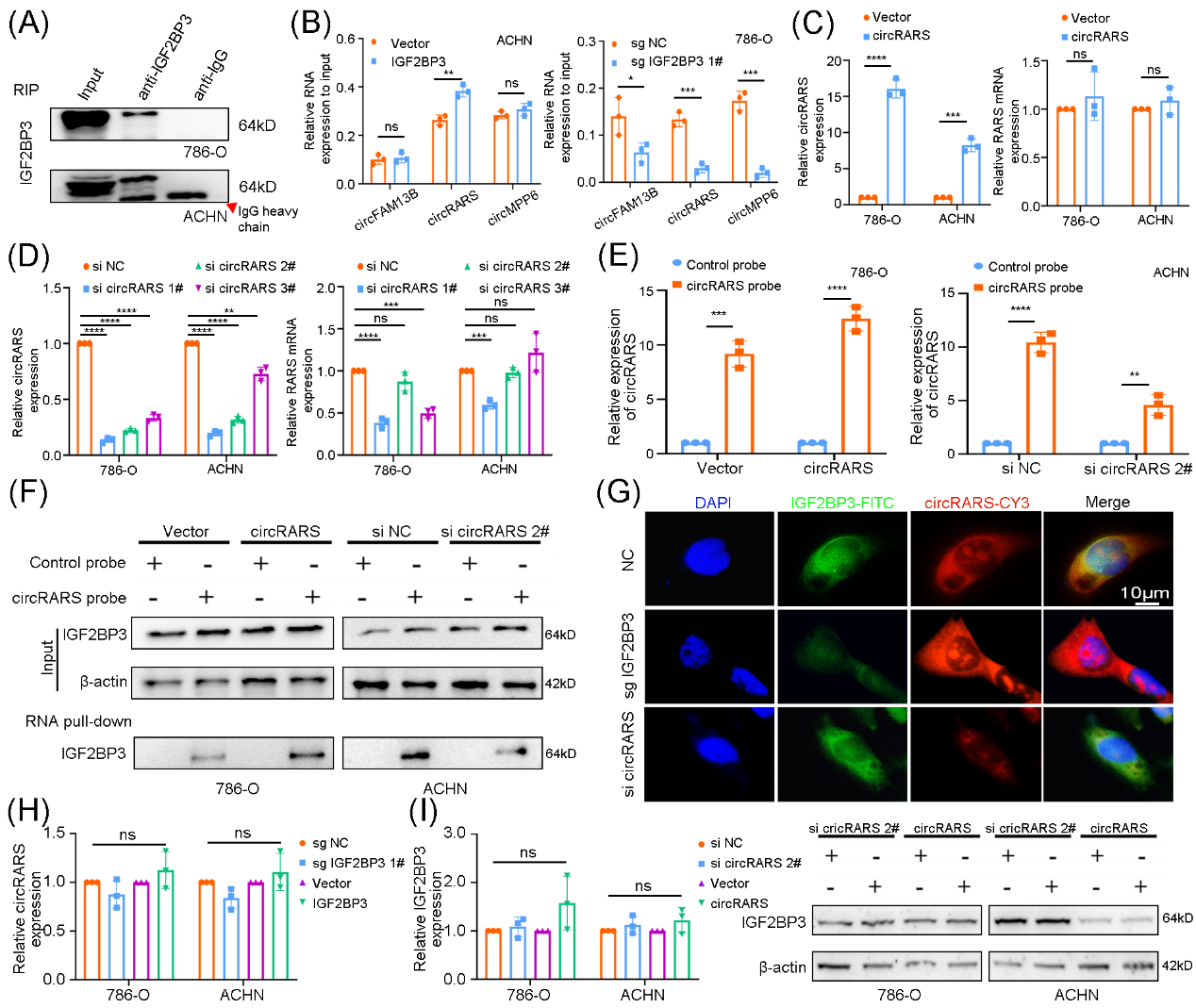


**Figure S4** IGF2BP3 binds circRARS to form a complex. **(A)** Western blotting assays showed the immunoprecipitation efficiency of IGF2BP3 antibody in RIP assays. **(B)** In IGF2BP3 knocked out or overexpressed RCC cells, RIP and qRT-PCR were used to evaluate the combination between IGF2BP3 and circRNAs (circFAM13B, circRARS and circMPP6). **(C-D)** qRT-PCR assays showed the expression of circRARS and mRNA of RARS after RCC cells transfected with circRARS overexpression vector **(C)** or circRARS siRNAs **(D)**. **(E)** In RCC cells, the RNA pulldown efficiency of biotin-labeled circRARS probe changed with circRARS knocked down or overexpressed. **(F)** Lysates of circRARS knocked down or overexpressed RCC cells were hybridized with biotin-labled circRARS probe and Western blotting showed the level of IGF2BP3 pulled down with circRARS probe. **(G)** Representative FISH and IF dual staining showed the expression level and localization of IGF2BP3 (green) and circRARS (red) in ACHN cells. Scale bars, 10 μm. **(H)** qRT-PCR assays showed the expression of circRARS in IGF2BP3 knocked out or overexpressed RCC cells. **(I)** qRT-PCR and Western blotting assays showed the expression of IGF2BP3 in circRARS knocked down or overexpressed RCC cells. *: P < 0.05, **: P < 0.01, ***P: < 0.001, ****P: < 0.0001. Error bars indicate mean ±SD.

**Figure S5.** Identification of circRARS.


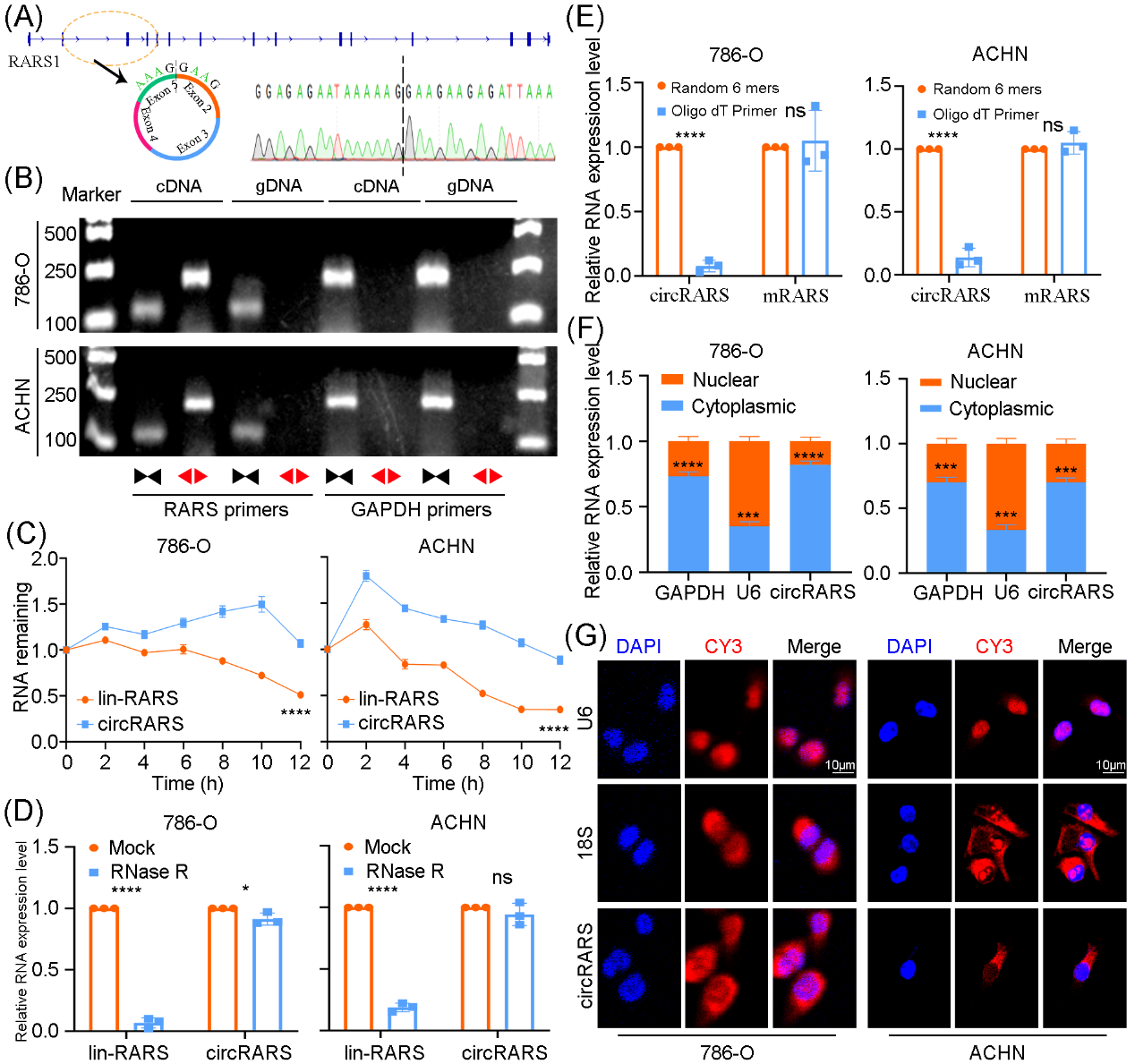


**Figure S5** Identification of circRARS. **(A)** Scheme illustrating the production of circRARs and sequencing analysis of head-to-tail splicing junction in circRARS. **(B)** The existence of circRARS was validated in RCC cells by qRT-PCR. Divergent primers amplified circRARS in cDNA but not genomic DNA (gDNA). GAPDH was used as negative control. Red arrows indicated divergent primers, and black arrows indicated convergent primers. **(C)** The relative RNA levels of circRARS and mRARS were analyzed by qRT-PCR after treatment with actinomycin D at the indicated time points in RCC cells. **(D)** The relative RNA levels were analyzed by qRT-PCR in RCC cells treated with or without RNase R. **(E)** Reverse transcription was performed by random 6 mers and oligo dT primer, respectively. Then, the relative RNA levels of circRARS and mRARS were analyzed by qRT-PCR. **(F)** Localization of IGF2BP3 was assessed by qRT-PCR in RCC cells. U6 and GAPDH were used as positive controls for nuclear RNA and cytoplasmic RNA, respectively. **(G)** The distribution of IGF2BP3 was analyzed by FISH in RCC cells. 18S and U6 showed cytoplasm and nucleus, respectively. Scale bars, 10 μm. *: P < 0.05, **: P < 0.01, ***P: < 0.001, ****P: < 0.0001. Error bars indicate mean ±SD.

**Figure S6.** circRARS is high-expressed and plays oncogenic roles in RCC.


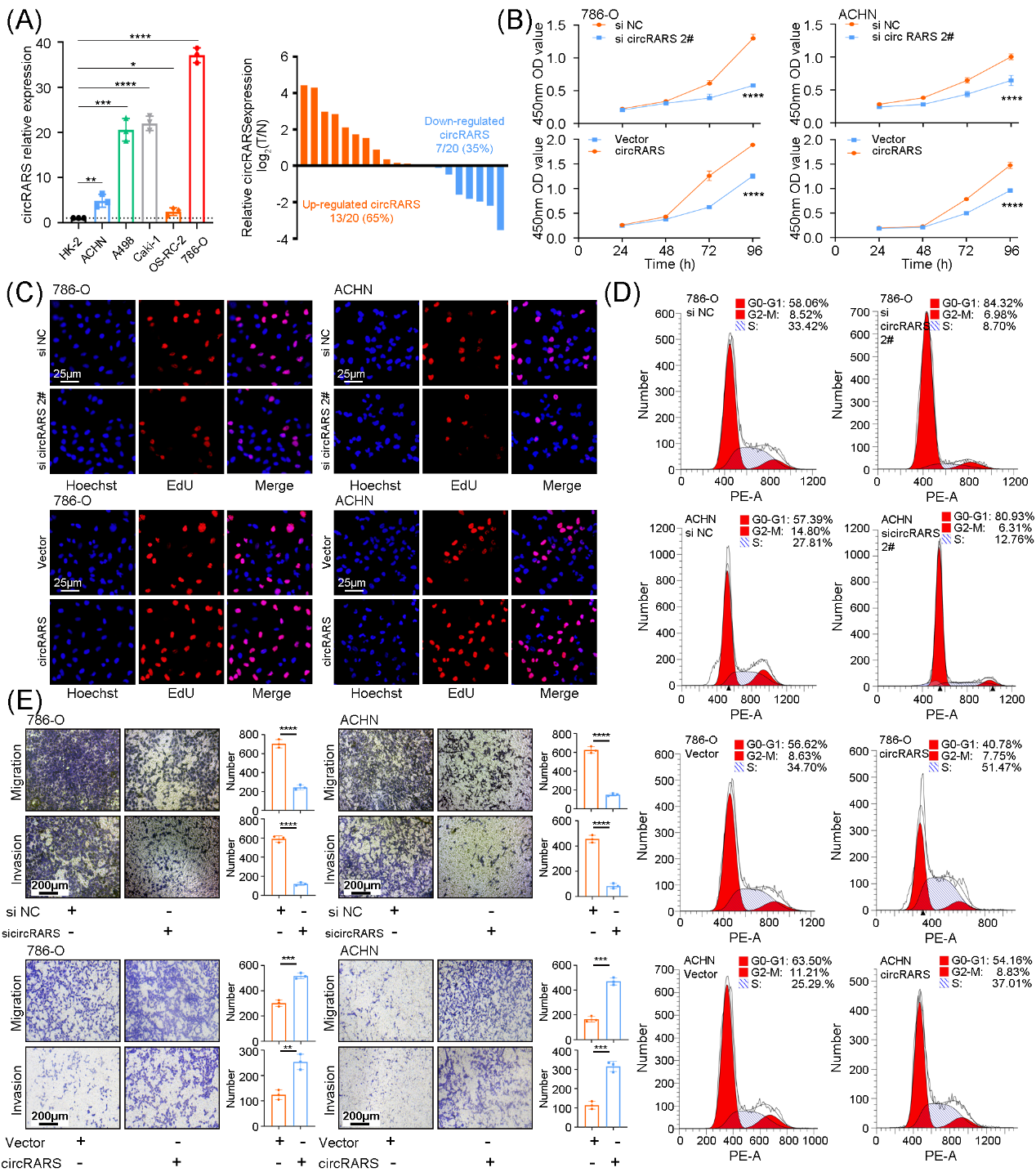


**Figure S6** circRARS is high-expressed and plays oncogenic roles in RCC. **(A)** The expression level of circRARS in RCC cell lines (ACHN, A498, Caki-1, OS-RC-2, and 786-O) and tissues. **(B-C)** RCC cells proliferation was evaluated with CCK8 assays **(B)** and EdU assays **(C)** after circRARS knocked down or overexpressed. Scale bars, 25 μm. **(D)** Cell cycle assays of RCC cells with circRARS knocked down or overexpressed. **(E)** Cell migration and invasion ability of circRARS knocked down or overexpressed RCC cells was measured with Transwell assays. Scale bars, 200 μm. *: P < 0.05, **: P < 0.01, ***P: < 0.001, ****P: < 0.0001. Error bars indicate mean ±SD.

**Figure S7.** IGF2BP3/circRARS complex accelerates the proliferation and metastasis of RCC.


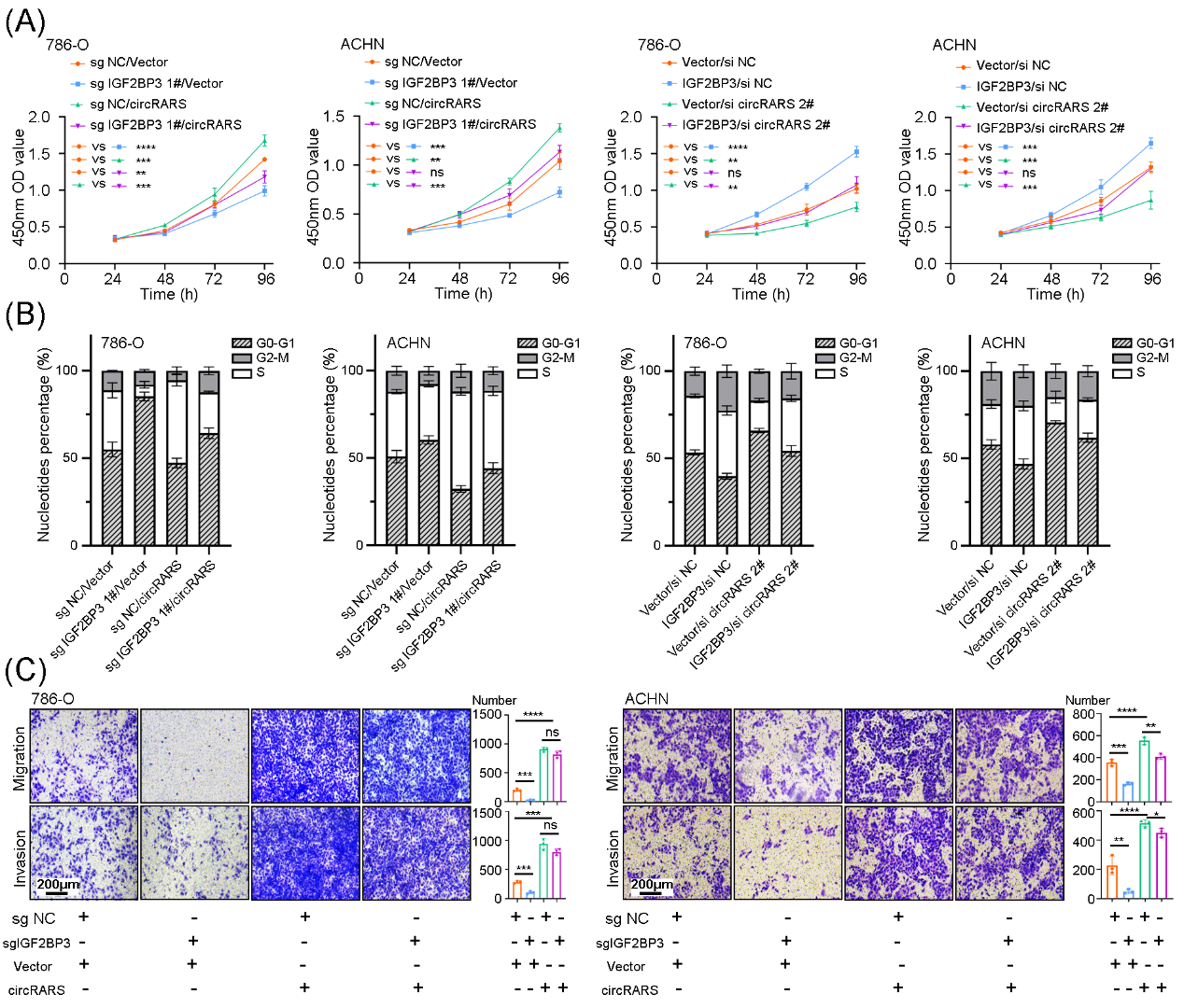


**Figure S7** IGF2BP3/circRARS complex accelerates the proliferation and metastasis of RCC. **(A)** RCC cells proliferation was evaluated with CCK8 assays under different treatment conditions. **(B)** Cell cycle assays of RCC cells under different treatment conditions. **(C)** Transwell assays measuring cell migration and invasion ability of RCC cells with IGF2BP3 knocked out and circRARS overexpressed. Scale bars, 200 μm. *: P < 0.05, **: P < 0.01, ***P: < 0.001, ****P: < 0.0001. Error bars indicate mean ±SD.

**Figure S8** IGF2BP3/circRARS complex regulates EMT signaling pathway in RCC cells.


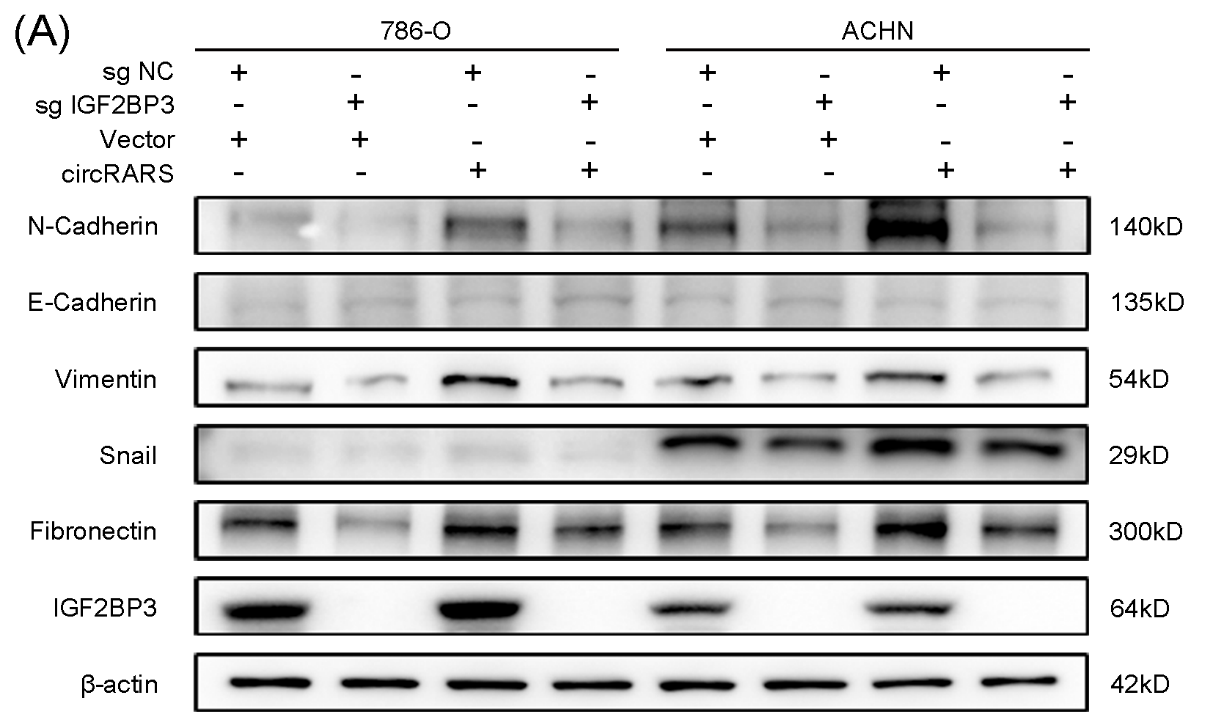


**Figure S8** IGF2BP3/circRARS complex regulates EMT signaling pathway in RCC cells. **(A)** Western blotting showed the expression level of EMT proteins including N-cadherin, E-cadherin, Vimentin, Snail and Fibronectin in RCC cells with IGF2BP3 knocked down and circRARS overexpression.

**Figure S9.** IGF2BP3/circRARS complex regulates downstream genes via affecting RNA stabilities.


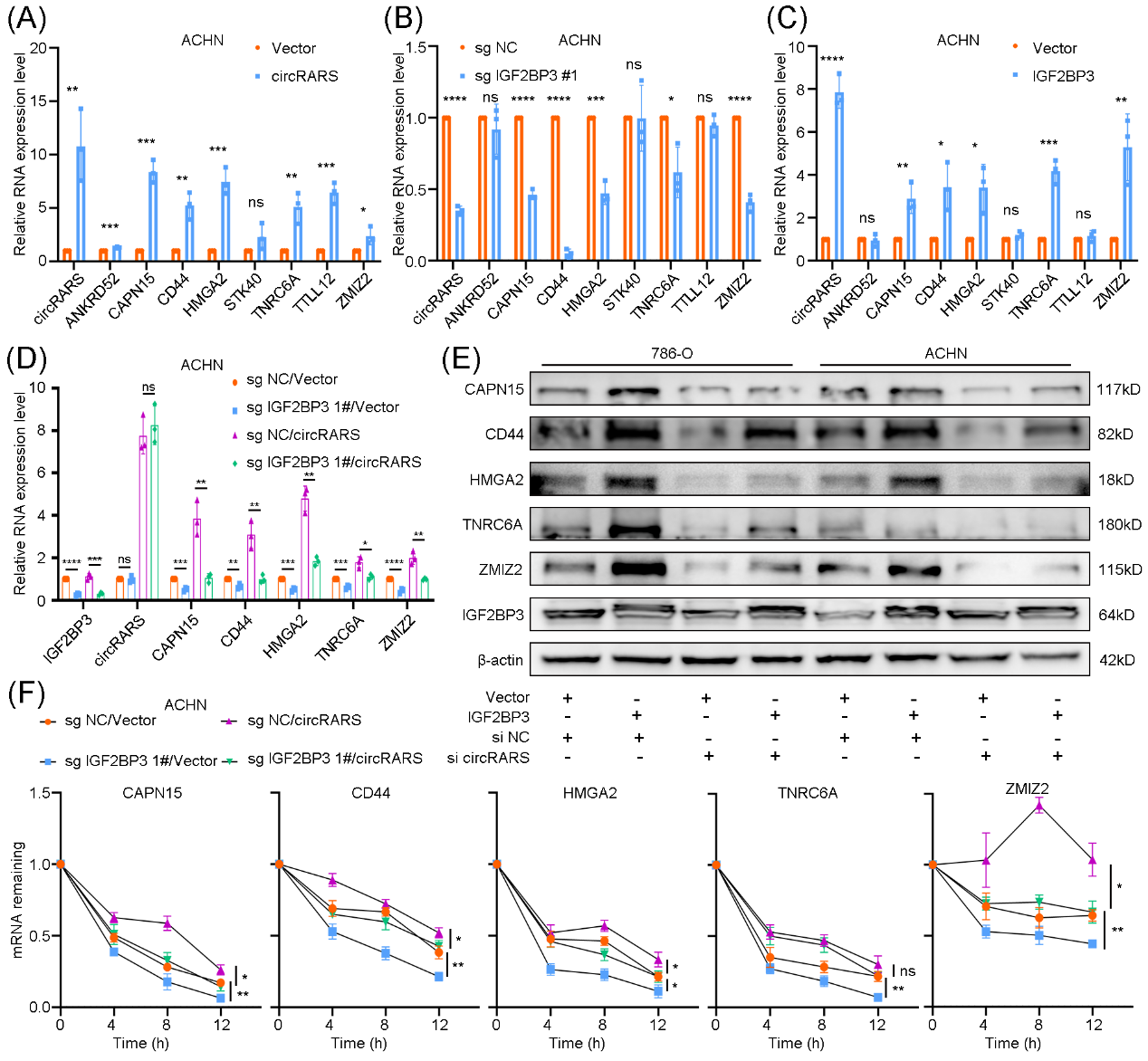


**Figure S9** IGF2BP3/circRARS complex regulates downstream genes via affecting RNA stabilities. **(A)** qRT-PCR was used to examine the expression level of downstream genes in circRARS overexpression ACHN cells. **(B-C)** qRT-PCR was used to examine the expression level of downstream genes in IGF2BP3 knocked-out **(B)** or overexpressed **(C)** ACHN cells. **(D)** qRT-PCR showed the downstream genes expression level in ACHN cells transfected with IGF2BP3 sgRNA or control, and co-transfected with vector or circRARS plasmid. **(E)** Western blotting showed the downstream proteins expression level in RCC cells transfected with IGF2BP3 overexpression or control vector, and co-transfected with circRARS siRNA or control. **(F)** The mRNA levels of downstream genes were measured by qRT-PCR in ACHN cells after treatment with actinomycin D at the indicated time points. *: P < 0.05, **: P < 0.01, ***P: < 0.001, ****P: < 0.0001. Error bars indicate mean ±SD.

**Figure S10.** Analysis of m6A modification sites of downstream targets.


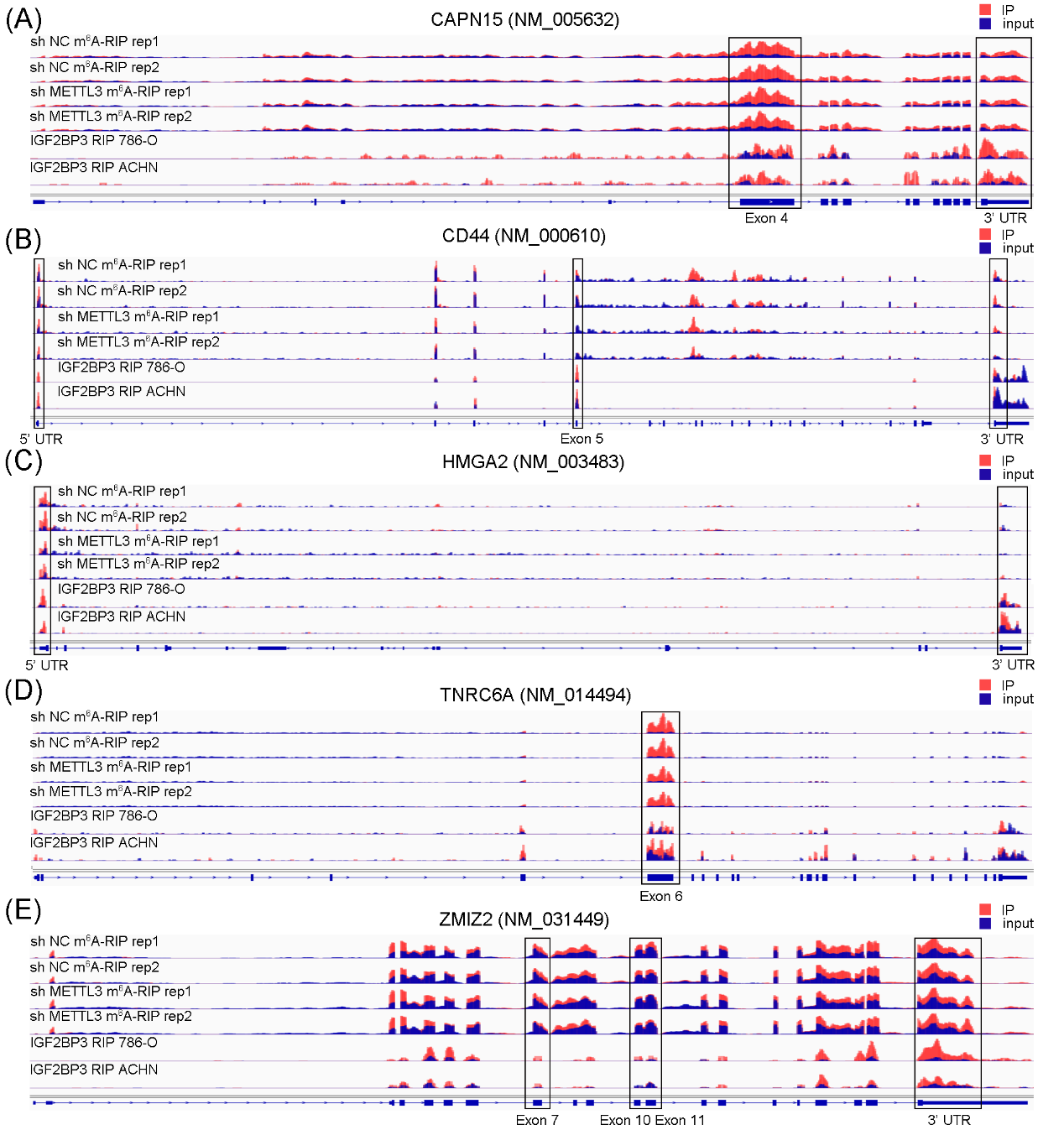


**Figure S10** Analysis of m6A modification sites of downstream targets. **(A-E)** Distributions of m^6^A peaks across the target transcripts based on m^6^A RIP- and IGF2BP3 RIP-sequencing data were analyzed with IGV software. The potential m^6^A-modified and IGF2BP3-binding regions in target transcripts were marked by rectangles.

**Figure S11.** circRARS assists IGF2BP3 in identification and combination of m^6^A modifications.


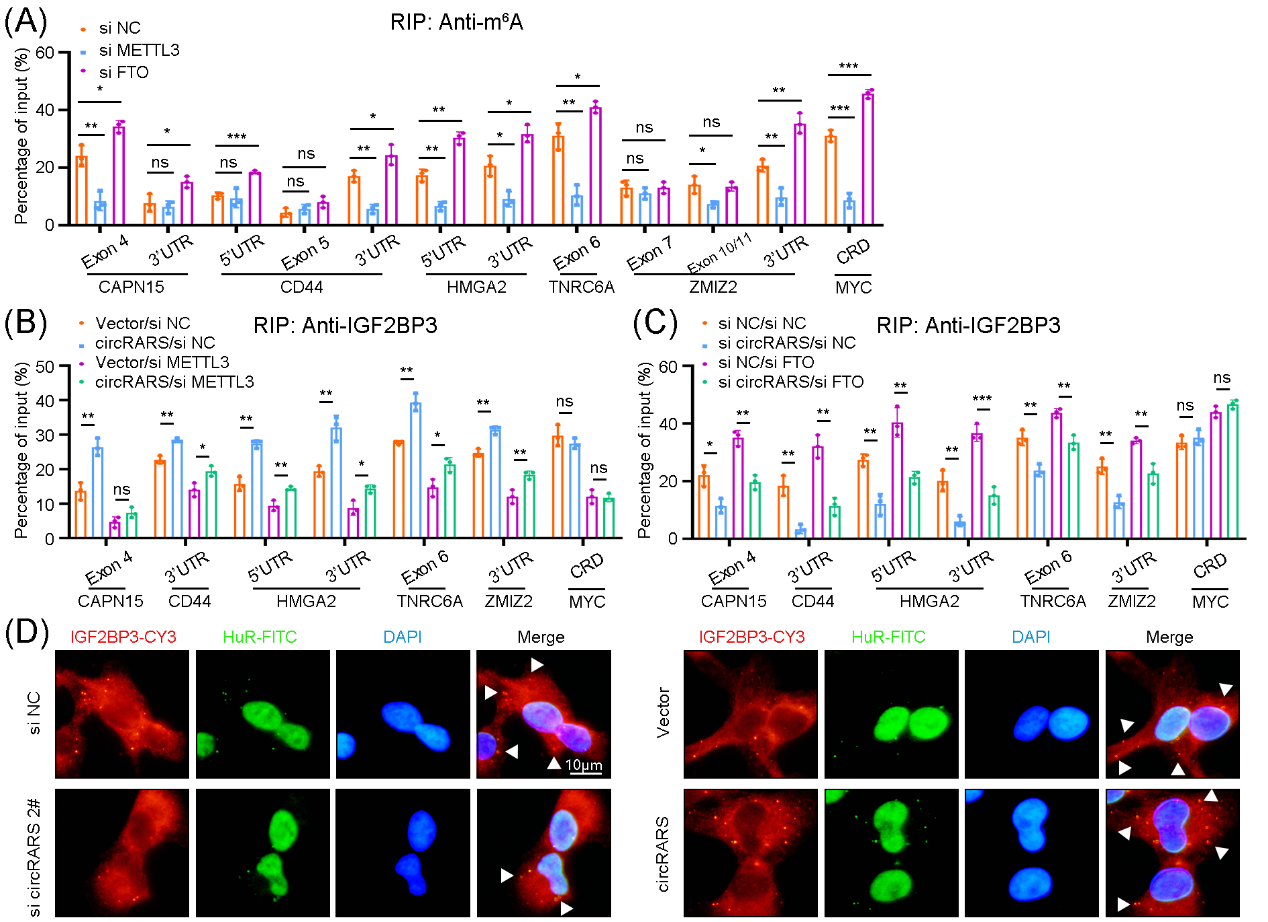


**Figure S11** circRARS assists IGF2BP3 in identification and combination of m^6^A modifications. **(A)** RIP targeted m^6^A and qRT-PCR assays showed the enrichment regions of m^6^A modifications in the mRNA of CAPN15, CD44, HMGA2, TNRC6A and ZMIZ2 in METTL3 or FTO knocked down ACHN cells. MYC CRD was used as a positive control. **(B-C)** RIP targeted IGF2BP3 and qRT-PCR assays showed the combination ability between IGF2BP3 and m^6^A modification regions of downstream genes in circRARS overexpressed and METTL3 knocked down **(B)** or circRARS knocked down and FTO knocked down **(C)** ACHN cells. **(D)** Co-localization of IGF2BP proteins with HuR in circRARS knocked down or overexpressed ACHN cells. Triangles indicated co-localization in cytoplasmic granules. Scale bars, 10 µm. *: P < 0.05, **: P < 0.01, ***P: < 0.001, ****P: < 0.0001. Error bars indicate mean ±SD.

**Figure S12.** The relationships between downstream targets expression and clinicopathological characteristics of RCC based on TCGA-KIRC dataset.


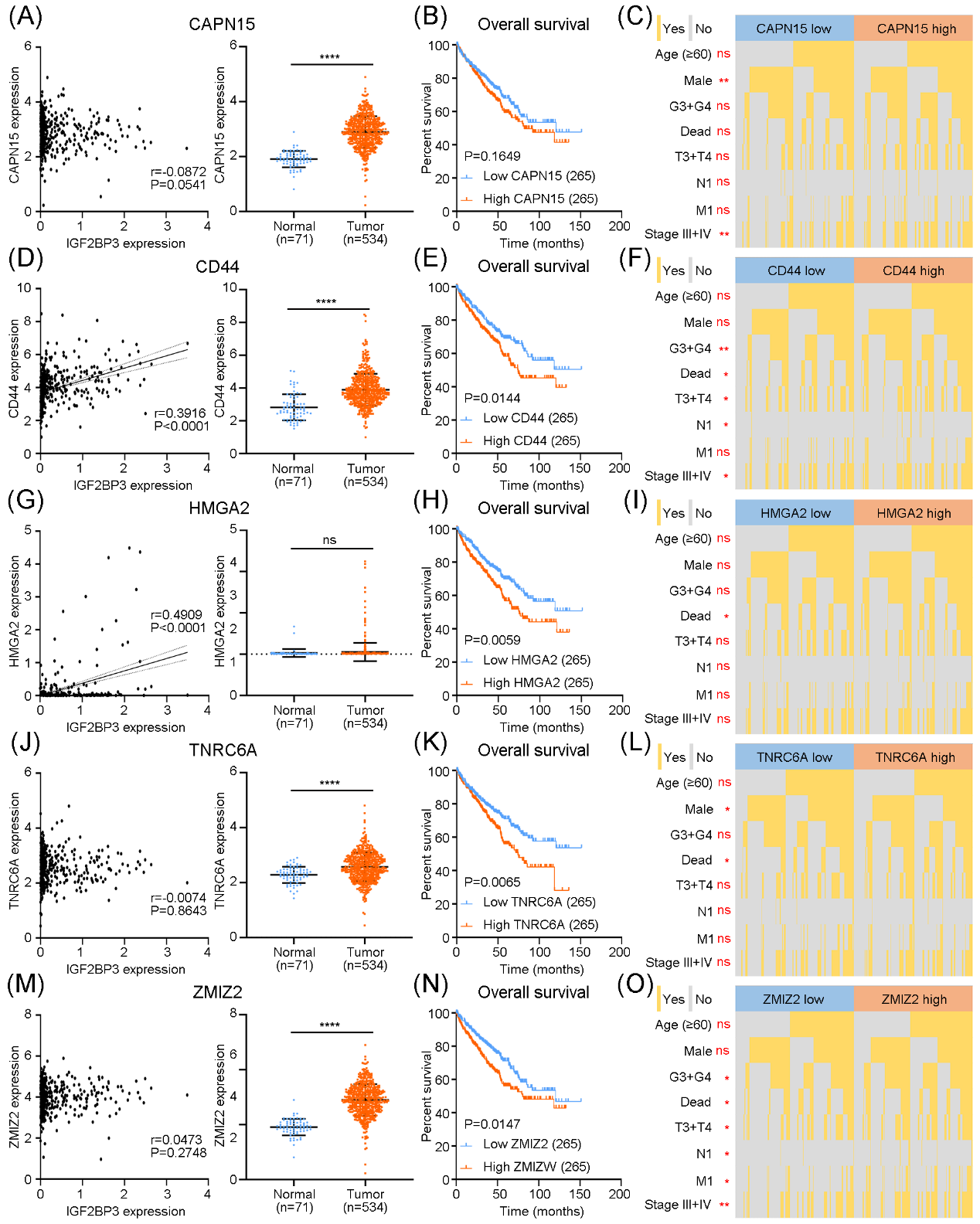


**Figure S12** The relationships between downstream targets expression and clinicopathological characteristics of RCC based on TCGA-KIRC dataset. The expression level analysis and correlation analysis with IGF2BP3 of **(A)** CAPN15, **(D)** CD44, **(G)** HMGA2, **(J)** TNRC6A, and **(M)** ZMIZ2 in RCC. The Kaplan–Meier survival analysis of **(B)** CAPN15, **(E)** CD44, **(H)** HMGA2, **(K)** TNRC6A, and **(N)** ZMIZ2 in RCC. Relationships between clinicopathological characteristics of RCC with **(C)** CAPN15, **(F)** CD44, **(I)** HMGA2, **(L)** TNRC6A, and **(O)** ZMIZ2. *: P < 0.05, **: P < 0.01, ***P: < 0.001, ****P: < 0.0001. Error bars indicate mean ±SD.

**Figure S13.** The expression of downstream targets clinical RCC specimens.


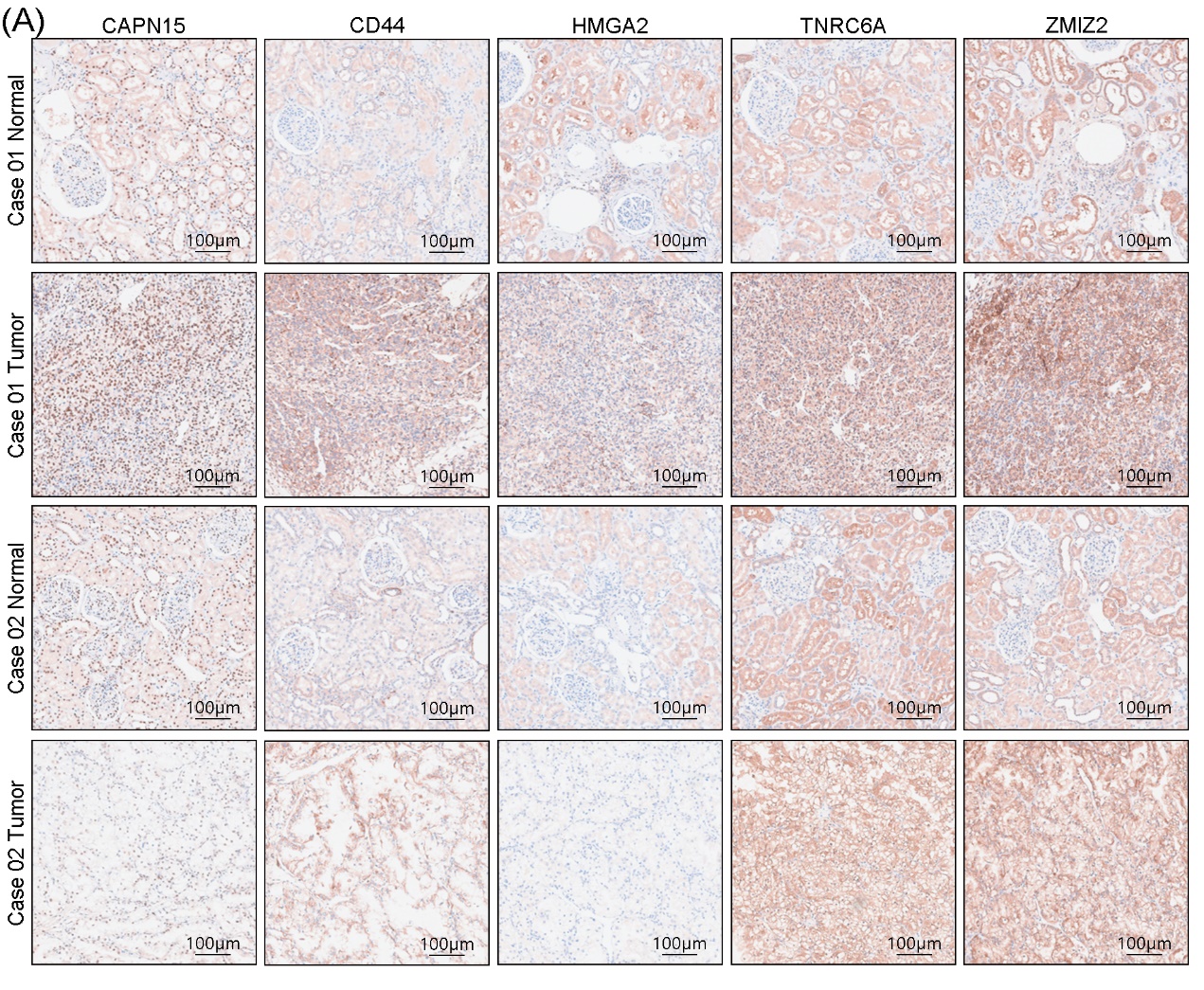
**Figure S13.** The expression of downstream targets clinical RCC specimens. (A) The immunohistochemistry staining of CAPN15, CD44, HMGA2, TNRC6A, and ZMIZ2 in clinical RCC specimens. Scale bars, 100 μm.

**Figure S14.** Interference efficiency of siRNAs.


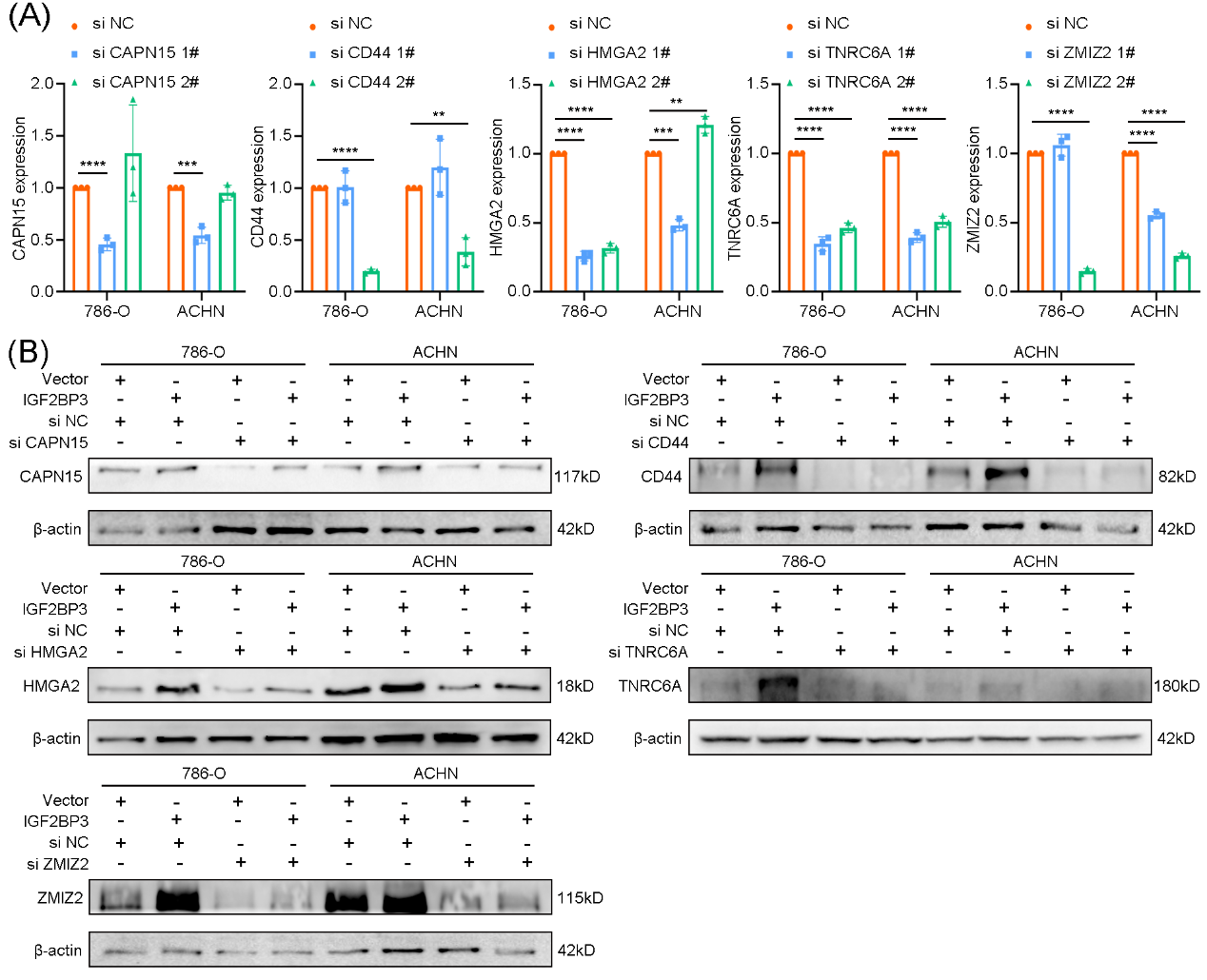


**Figure S14** Interference efficiency of siRNAs. **(A)** qRT-PCR showed mRNA expression level in RCC cells transfected with siRNA. **(B)** Western blotting assays showed protein levels of downstream targets in IGF2BP3 overexpressed RCC cells co-transfected with different siRNAs. *: P < 0.05, **: P < 0.01, ***P: < 0.001, ****P: < 0.0001. Error bars indicate mean ±SD.

**Figure S15.** IGF2BP3/circRARS complex performs biological functions partially through downstream proteins.


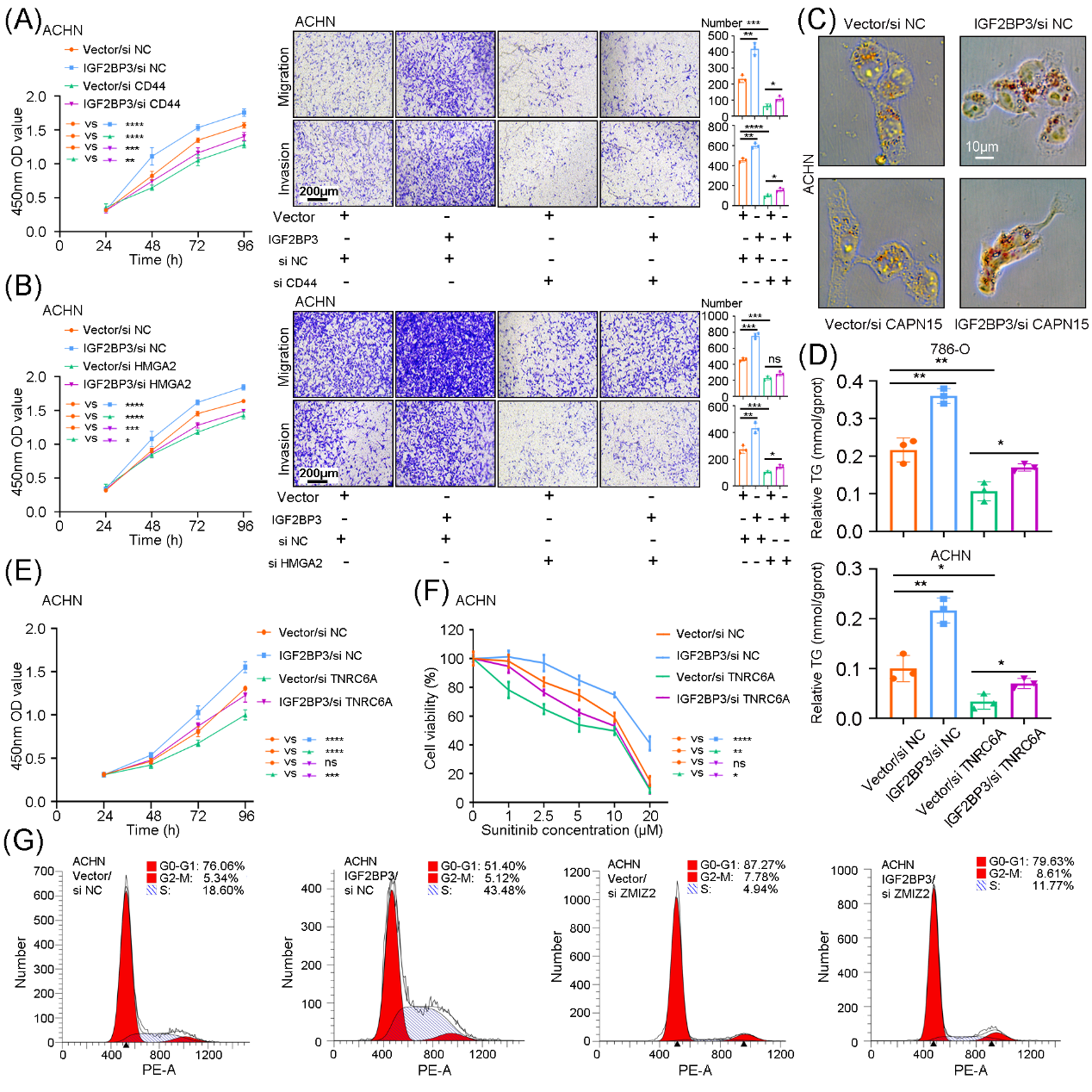


**Figure S15** IGF2BP3/circRARS complex performs biological functions partially through downstream proteins. **(A)** Proliferation and metastasis were evaluated with CCK8 assays and Transwell assays of IGF2BP3 overexpression ACHN cells transiently transfected with CD44 siRNA or control**.** Scale bars, 200 μm. **(B)** Proliferation and metastasis were evaluated with CCK8 assays and Transwell assays of IGF2BP3 overexpression ACHN cells transiently transfected with HMGA2 siRNA or control**.** Scale bars, 200 μm. **(C)** Representative Oil Red O stains of IGF2BP3 overexpression ACHN cells transiently transfected with CAPN15 siRNA or control**.** Scale bars, 10 μm. **(D)** Relative TG (mmol/gprot) levels in RCC cells with IGF2BP3 overexpression and CAPN15 knocked down assessed by a triglyceride assay kit. **(E)** Proliferation was evaluated with CCK8 assays of IGF2BP3 overexpression ACHN cells transiently transfected with TNRC6A siRNA or control**. (F)** Cell sensitivity to sunitinib was measured via CCK8 assays of IGF2BP3 overexpressed and TNRC6A knocked down ACHN cells treated with sunitinib in different concentration**. (G)** Cell cycle assays of IGF2BP3 overexpression ACHN cells transiently transfected with ZMIZ2 siRNA or control. *: P < 0.05, **: P < 0.01, ***P: < 0.001, ****P: < 0.0001. Error bars indicate mean ±SD.

**Figure S16.** Expression level of cancer stemness marker and downstream proteins and in renal orthotopic xenografts of nude mice.


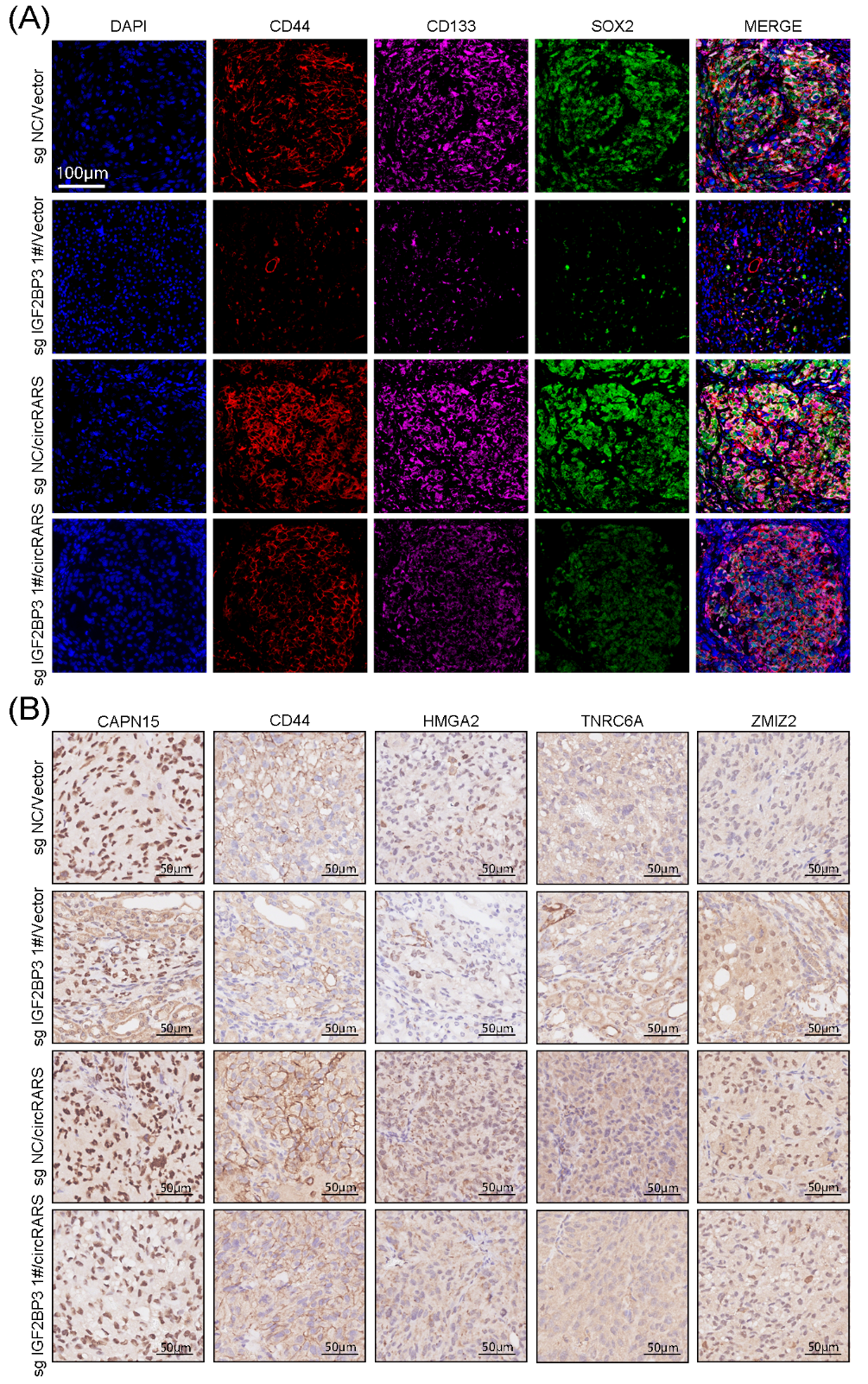


**Figure S16** Expression level of cancer stemness marker and downstream proteins in renal orthotopic xenografts of nude mice. **(A)** IF images of CD44, CD133, and SOX2 in renal orthotopic xenografts of nude mice under various treatment conditions. Scale bars, 100µm. **(B)** IHC assays showed expression level of CAPN15, CD44, HMGA2, TNRC6A, and ZMIZ2 in renal orthotopic xenografts of nude mice under various treatment conditions. Scale bars, 50 µm.
